# From regulation to deregulation of p53 in hematologic malignancies: implications for diagnosis, prognosis and therapy

**DOI:** 10.1186/s40364-024-00676-9

**Published:** 2024-11-14

**Authors:** Seyed Esmaeil Ahmadi, Elahe Rahimian, Samira Rahimi, Bahman Zarandi, Mehran Bahraini, Maral Soleymani, Seyed Mehrab Safdari, Ashkan Shabannezhad, Niloofar Jaafari, Majid Safa

**Affiliations:** 1 Department of Hematology and Blood Banking, Faculty of Allied Medicine, Iran University of Medical Sciences, Tehran, Iran; 2 Department of Medical Translational Oncology, National Center for Tumor Diseases (NCT) Dresden, Dresden, Germany; 3 Faculty of Medicine, Ahvaz Jundishapur University of Medical Sciences, Ahvaz, Iran; 4 Department of Pharmacology and Chemical Biology, University of Pittsburgh, Pittsburgh, PA 15261 USA

**Keywords:** P53, Tumor suppressor, P53 Regulation, DNA damage response, Prognosis, Diagnosis, Therapy, Hematological malignancies

## Abstract

The p53 protein, encoded by the *TP53* gene, serves as a critical tumor suppressor, playing a vital role in maintaining genomic stability and regulating cellular responses to stress. Dysregulation of p53 is frequently observed in hematological malignancies, significantly impacting disease progression and patient outcomes. This review aims to examine the regulatory mechanisms of p53, the implications of *TP53* mutations in various hematological cancers, and emerging therapeutic strategies targeting p53. We conducted a comprehensive literature review to synthesize recent findings related to p53's multifaceted role in hematologic cancers, focusing on its regulatory pathways and therapeutic potential. *TP53* mutations in hematological malignancies often lead to treatment resistance and poor prognosis. Current therapeutic strategies, including p53 reactivation and gene therapy, show promise in improving treatment outcomes. Understanding the intricacies of p53 regulation and the consequences of its mutations is essential for developing effective diagnostic and therapeutic strategies in hematological malignancies, ultimately enhancing patient care and survival.

## Introduction

The p53 protein, encoded by the *TP53* gene, was first discovered in 1979. It was identified as a tumor suppressor in 1989. Although there are various tumor suppressors, p53 is known as the “guardian of the genome” [1, 2]. It has an indispensable role in directing cell fate during cellular stresses, such as hypoxia, oncogene activation, metabolic dilemma, ribosomal stress, and genotoxicity [3, 4]. The activation of p53 occurs as a result of DNA damage responses (DDRs), which can lead to cell cycle arrest, DNA repair, senescence, or programmed cell death (e.g., apoptosis and ferroptosis) [5, 6]. The human *TP53* gene is mutated in approximately 50 to 60% of human cancers (more than 80% of which are missense mutations) [7]. In fact, *TP53* has been designated as the most frequently scrutinized gene of all time [8].

The p53 protein harbors five main domains: (1) the N-terminal domain, (2) the central DNA-binding domain (DBD) that directly facilitates the binding with DNA, (3) the oligomerization domain forming the tetramerization of p53 protein, (4) the proline-rich domain which is involved in maintaining the stability of the protein, and (5) the C-terminal domain that has regulatory sites for both the DNA-specific and nonspecific p53 binding and also senses the damaged DNA [9, 10]. While tumor suppressors are usually inactivated by frameshift or nonsense mutations, the most frequent mechanisms by which the wild-type p53 is inactivated in primary human tumors are missense mutations in the coding region with a strong predominance for exons 4–9; therefore, most studies have focused on these regions [11].

The mutated *TP53* at the germline level predisposes patients to a condition known as Li–Li-Fraumeni syndrome [12]. *TP53* is frequently mutated in a variety of human cancers, and hematological neoplasms, including myelodysplastic syndrome (MDS), multiple myeloma (MM), acute and chronic leukemias (e.g., AML, ALL, CML, CLL), and lymphomas are not exceptions [13–18]. Inhibition of p53 and the mutant forms are associated with leukemia resistance to treatments and poor prognosis [19, 20]. Additionally, mutation or deletion in *TP53* might cause progression in leukemia via aberrant self-renewal [21]. The emergence of advanced technologies such as next-generation sequencing (NGS) and gene-editing-based techniques such as CRISPR\Cas and gene delivery systems have paved the way for overcoming the p53-involved disorders [22, 23].

Complex conditions of cancers like leukemias demand a precise approach toward its management and treatment**.** In cases of hematological malignancies, restoring the activity of p53 and its tumor-suppressive effects by small molecules have shown to be effective [24–26]. Not only does reactivating wild-type (wt) p53 have an influential role in the p53 restoration, but also stabilizing p53, for instance, using MDM2 inhibitors in wt p53-expressing cancer cells, has shown promising results. [27, 28]. Furthermore, gene therapy approaches like an adenoviral-based gene therapy drug named Gendicine (recombinant human p53 adenovirus) became the first approved gene therapy for p53 restoration by the China Food and Drug Administration (CFDA) [29].

*TP53* dysregulation and alteration can increase the risk of various types of cancers, including hematological malignancies (HMs). *TP53* missense mutations and 17p deletion are commonly seen in HM patients, with the absence or presence of mutant p53 associated with chemoresistance, relapse, and shorter patient survival. Determining *TP53* status could thus be beneficial for diagnosis and prognosis evaluation for HMs and for choosing suitable potential therapeutic approaches such as p53 reactivation stabilization or gene therapy. This article addresses the importance of p53 in hematologic cancers as a diagnostic, prognostic, and therapeutic factor.

## p53 regulation

In the absence of any stimuli or stress, repressive factors, especially MDM2, are responsible for keeping p53 levels low, although following DDRs, p53 becomes phosphorylated and immune to the MDM2 restraining effect. [30]. In addition to MDM2-mediated regulation, the regulation of p53 takes place by other tortuous mechanisms. Whether cells are in non-stressed or stressed conditions, p53’s fate is directed in a multi-dimensional process through transcriptional, post-transcriptional, and post-translational regulation [3, 13].

### Transcriptional regulation of p53

The regulation of the p53 gene is a complex process influenced by various transcription factors (TFs) that play a pivotal role in modulating its expression. Understanding these interactions is crucial for comprehending how p53 functions as a tumor suppressor under normal and stressed conditions. Several TFs are directly involved in p53 regulation, including the Myc/Max heterodimer, AP1, NF-κB, and USF1 [31–33].

The AP1 family, which includes members like FUS and JUN, has diverse roles in cellular processes such as proliferation, differentiation, apoptosis, and survival. Interestingly, the impact of AP1 can be dual-sided; for instance, c-Fos activity has been linked to both tumor-suppressive and tumorigenic outcomes, depending on the cellular context [34]. On the other hand, the c-Myc protein, in conjunction with its co-factor Max, has been shown to upregulate p53 expression, promoting apoptosis in response to cellular stress [35]. Contrastingly, the Myc-associated zinc finger protein (MAZ) typically suppresses the TP53 promoter, but it detaches upon activation by Akt signaling pathways, influenced by factors such as EGF or Her2 receptors [36].

Additional TFs, like Yin-Yang (YY)-1 [37] and nuclear factor 1 (NF1) [38], exert a positive regulatory effect on TP53 expression, although their activity is often tissue-specific. Interestingly, YY1 displays both oncogenic and tumor-suppressive properties, with its effects varying significantly depending on the cancer cell type. The ETS family of proto-oncogenes also plays a notable role, with members that either activate or suppress the TP53 promoter depending on the specific context [39, 40].

These regulatory mechanisms highlight the intricate control network governing p53 expression, underscoring its sensitivity to both internal cellular signals and external stressors. Understanding these TFs' roles is critical for developing targeted therapies that can manipulate p53 activity in cancer cells.

### Post-transcriptional regulation of p53

Beyond transcriptional control, the p53 gene is also subject to complex post-transcriptional regulation that significantly impacts its stability and function. This layer of regulation involves interactions between the p53 mRNA and various non-coding RNAs, proteins, and other factors that modulate its expression. The 5' and 3' untranslated regions (UTRs) of p53 mRNA are crucial in this regulatory process, where mutations in these areas can disrupt the binding of essential trans-acting elements [41].

A specific cis-acting restrainer element in the 3' UTR of p53 mRNA plays a role in maintaining its regulation under normal conditions. However, following cellular stress such as irradiation, this restrainer element weakens, leading to increased interactions between p53 mRNA and polysomes, thereby enhancing protein synthesis [42].

Post-transcriptional regulators of p53 can be broadly classified into positive and negative regulators. Positive regulators, including proteins like HuR, RBM38, Hzf, Wig-1, and several others, promote p53 stability and function. In contrast, negative regulators, such as lncRNA 7SL, miRNA-125b, and others, act to inhibit p53 expression and activity. Each of these factors plays a vital role in fine-tuning the cellular response to DNA damage and stress, as detailed in Table 1.

**Table 1:** Post-transcriptional regulators of p53

Role	RNA binding factors	Name	Description/Function	Ref
**Positive regulators**	**HuR**	Human antigen R	Intensifies p53 translation, stabilizes p53 mRNA in response to UV irradiation	[43]
	**RBM38**	RNA binding motif protein 38	RBM38 phosphorylated form promotes p53 translation	[44, 45]
	**Hzf**	Hematopoietic zinc finger protein	Enhancing p53 translation with HuR cooperation as a result of p19 (ARF) signals	[46]
	**Wig-1**	Wild-type p53-induced gene 1	Stabilize p53 mRNA	[47, 48]
	**CPEB1**	Cytoplasmic polyadenylation element-binding protein 1	It recruits the GLD-4 to boost up polyadenylation of p53 mRNA	[49]
	**PTB**	Tract binding protein	PTB positively regulates the expression of p53 and its isoform Δ40p53 α	[50]
	**GSK3**	Glycogen synthase kinase 3	Phosphorylates RBM38 at Ser195 to repress its inhibitory effect on p53 translation	[51]
	**RPL26**	Ribosomal protein L26	Enhances p53 translation by binding to p53 5′UTR	[52]
	**Wrap53**	WD repeat-containing antisense to p53	Overexpression of Wrap53 results in a higher level of p53 mRNA	[53]
	**DAP5**	Death associate protein 5	Promote p53/47 expression	[54]
	**TCP80**	Translational control protein 80	Overexpression of TCP80 along with RHA promotes p53 translation	[55]
	**RHA**	RNA helicase A	Promotes p53 translation	[55]
	**hnRNP L**	Heterogeneous nuclear ribonucleoprotein L	Deletion of hnRNP L gene showed a low level of p53	[56]
	**hnRNP Q**	Heterogeneous nuclear ribonucleoprotein Q	Enhances p53 translation and apoptosis progression	[57]
	**PSF/SFPQ**	Splicing factor	Promote p53 and p53/47 expression	[58]
	**Annexin A2**	–-	Enhances p53 translation in a Ca_2_^+^ dependent following ER-induced stress	[58]
	**MDM2**	Murine double minute 2	Promote p53 translation	[59]
	**APP**	Amyloid precursor protein	Intensifies p53/47 expression	[60]
**Negative regulators**	**lncRNA 7SL**	Long non-coding RNA 7SL	Competes with HuR to suppress p53 translation	[61]
	**RBM38 (RNPC1)**	RNA binding motif protein 38	RBM38 inhibits p53 translation, but its phosphorylated form promotes p53 translation	[44, 45, 52]
	**TS**	Thymidylate synthase	Suppresses p53 translation	[62, 63]
	**Tai-1**	Cytotoxic granule-associated RNA binding protein	Repressed p53 mRNA in stress granules in normal condition	[64]
	**p53**	Cellular tumor antigen p53	p53 Repress its mRNA by binding to the 5′ UTR	[65]
	**Nucleolin**	–-	Overexpression of Nucleolin represses p53 translation	[52]
	**Pdcd4**	Programmed cell death 4	Interacts with eIF4A and inhibits p53 translation	[66]
	**miRNA-125b**	–-	Compete with HuR to repress the p53 translation. Also, halts HuR	[67, 68]
	**TRIM21**	Tripartite motif-containing protein 21	Inhibits HuR in response to UV irradiation	[68]
	**PARN**	Poly(A)-specific ribonuclease	Deadenylase the p53 mRNA and destabilized it, and also indirectly repress p53 translation	[69, 70]

The balance between these positive and negative regulators determines the outcome of p53 activity, which has profound implications for cell fate, especially in cancer. Understanding this regulatory landscape offers valuable insights into potential therapeutic targets that could enhance p53's tumor-suppressive functions while minimizing its negative effects on cancer progression.

### Post-translational regulation of p53

**Fig. 1:**
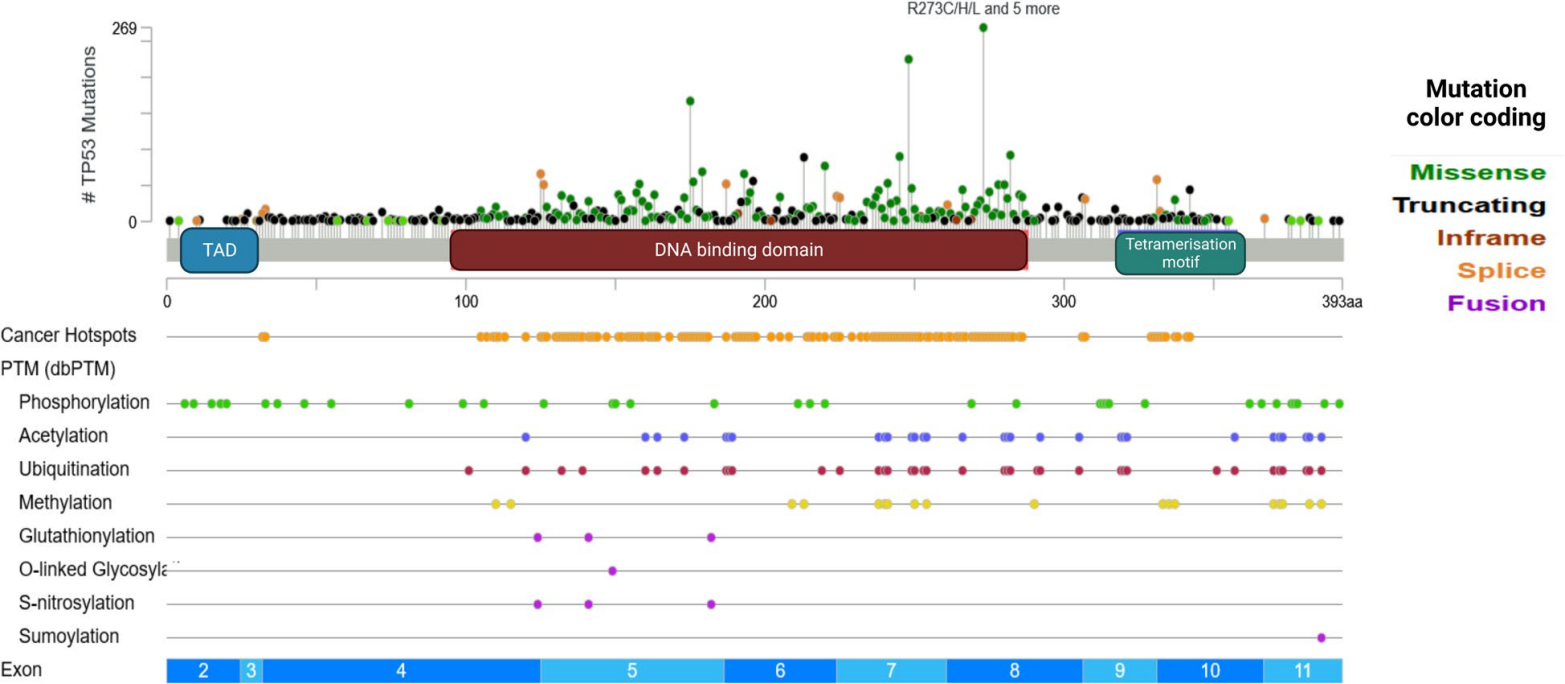
Map of mutations and PTMs of p53 generated using cBioPortal tools (http://www.cBioPortal.org) and curated manually

The p53 protein undergoes post-translational modifications (PTMs) at its five discrete domains, namely transactivation domains (TADs), proline-rich domain (PRD), DBD, the tetramerization domain (TD), and carboxy-terminal domain (CTD). The p53 domains harbor specific sites, which PTMs such as ubiquitination, SUMOylation, NEDDylation, methylation, acetylation, and phosphorylation can occur upon, either in stressed or non-stressed conditions (Fig. 1) [71].

Mediators involved in ubiquitination, such as MDM2, MKRN1, MSL2, and E4F1, exhibit distinct functionalities; MDM2 and MKRN1 are associated with proteasomal degradation, MSL2 plays a role in nuclear export, and E4F1 is implicated in inducing cell cycle arrest specifically at the G0/G1 phase [72–74]. SUMOylation is an additional post-translational process that regulates p53. The key mediator involved in this regulation is known as PIASs, which function to suppress p300-induced acetylation while concurrently deactivating the transcriptional activity of p53 [75]. The mediators MDM2 and FOXO11, known for their involvement in NEDDylation, act to suppress the transcriptional activity associated with p53 activation [76, 77]. Various factors can modulate the activity of p53 by phosphorylating the mediators within the p53 regulation pathway. These factors contribute to processes such as DNA repair, cell cycle regulation, and apoptosis. The pathway involves key players including ChK1/2, ATM, CK1, ATR, and HIPK2. ChK1/2 specifically regulates DNA repair and the cell cycle regulation, ATM and ATR influence P53 via cell cycle regulation, CK1 modulates P53 by acting upon MDM2, and HIPK2 regulates P53 activity within the context of P53 apoptosis regulation [74, 78–80].

NAD(P)H:quinone oxidoreductase 1 (NQO1) plays a crucial role in the regulation of p53 in cancer, primarily by stabilizing p53 and preventing its degradation through the ubiquitin-independent 20S proteasome pathway. NQO1 expression is upregulated during oncogene-induced senescence (OIS), where it promotes p53 accumulation, reinforcing the senescence phenotype and acting as a barrier to cell transformation and tumorigenesis [81]. The NRF2/KEAP1 signaling pathway regulates NQO1 during OIS, and its depletion delays senescence onset, facilitating cancer progression. Furthermore, in chronic lymphocytic leukemia (CLL) [82], NQO1 gene variability has been associated with cytogenetic abnormalities and TP53 disruptions, suggesting its involvement in p53 pathway integrity. Additionally, in hepatocellular carcinoma (HCC), NQO1 promotes cell proliferation and metastasis through the NQO1/p53/SREBP1 axis, which regulates lipid metabolism and the epithelial-to-mesenchymal transition (EMT). Together, these findings underscore NQO1's significance in maintaining p53 function, contributing to tumor suppression, and influencing cancer progression [83].

Another regulatory mechanism post-translationally affecting p53 is acetylation. P300, PCAF, TIP60, MOF, and MOZ serve as crucial mediators within this pathway, with P300 exhibiting diverse functions and significant importance. Specifically, MOF and TIP60 facilitate apoptosis, MOZ halts cell cycle progression, PCAF inhibits apoptosis (displaying contrasting roles to MOF and TIP60), and P300 governs cell cycle regulation, expression of proapoptotic genes, thereby enhancing apoptosis, and ultimately modulates P53 activity [84–86]. Another post-translational process that governs the activity of p53 is known as methylation. Key players in this pathway include SMYD2, SETD7, G9a/GLP, and SETD8. SMYD2 and SETD8 are responsible for inhibiting the interaction between p53 and the CDKN1A promoter, while SETD7 can both enhance the stability of p53 and trigger programmed cell death, and G9a/GLP contributes to the suppression of p53 [87–90].

Furthermore, other factors that play a part in PTM have been mentioned in Table 2. All in all, PTMs have shown a broad spectrum of regulatory effects on p53, demonstrating the tortuous complexity of p53 regulation, which calls for further investigation into how PTMs orchestrate p53 functions.

**Table 2:** Post-translational modifications of p53

Modification type	Mediator	Modification site(s)	Effect	Ref
**Ubiquitination**	MDM2	Lys370-Lys372-Lys373-Lys381-Lys382-Lys386	Proteasomal degradation	[91]
	MKRN1	Lys291-Lys292	Proteasomal degradation	[92]
	MSL2	Lys351-Lys357	Nuclear Export	[73]
	E4F1	Lys320	Cell cycle arrest at G0/G1	[74]
**SUMOylation**	PIASs	Lys386	Inhibiting p300-mediated acetylation and inactivating p53 transcription	[75]
**NEDDylation**	MDM2	Lys370-Lys372-Lys373	Hindering transcriptional activation activity of p53	[77]
	FOXO11	Lys320 and Lys321	Hindering transcriptional activation activity of p53	[76]
**Phosphorylation**	ChK1/2	Ser6-Ser15-Ser20-Ser33-Ser37-Ser313-Ser 314-Ser366-Ser378-Thr18-Thr387	(1) Higher p53 transcription activity and reduced MDM2 inclination to p53, followed by cell-cycle regulation and DNA repair (2) Enhances further acetylation by KATs	[74, 93–99]
	ATM	Ser9-Ser15-Ser46	Mainly, regulates p53 toward cell-cycle regulation	[100]
	CK1	Thr18	Directly blocks MDM2 binding to p53	[79]
	ATR	Ser15	Regulates p53 toward cell-cycle arrest	[80]
	HIPK2	Ser46	Regulates p53 toward apoptosis	[78]
**Acetylation**	p300	Lys370-Lys372-Lys373	Activation of CDKN1A (encoding p21) and cell-cycle regulation	[101, 102]
		Lys320-Lys382	Cell-cycle arrest by activating transcription of CDKN1A, and enhancing apoptosis	[103]
		Lys381-Lys382	Promote transcription of pro-apoptotic gene (*PUMA)*	[104]
		Lys164	Changing p53 conformation and leading to interaction with its co-activators	[105]
		Lys305	Promotes transcriptional activity of p53	[106]
	PCAF	Lys317-Lys320	Suppressing apoptosis, and providing time for DNA repair	[107, 108]
	TIP60	Lys120	Promoting apoptosis	[86]
	MOF	Lys120	Promoting apoptosis	[84]
	MOZ	Lys120	Cell-cycle arrest	[85]
**Methylation**	SMYD2	Lys370	Monomethylation hinders p53 binding to *CDKN1A* promoter	[89]
	SETD7	Lys372	Monomethylation and Dimethylation promote p53 stability and apoptosis	[87, 89]
	G9a/GLP	Lys373	Dimethylation causes p53 repression	[88]
	SETD8	Lys 382	Monomethylation hinders p53 binding to *CDKN1A* promoter	[90]

## The effects of p53 status on cancer

**Fig. 2:**
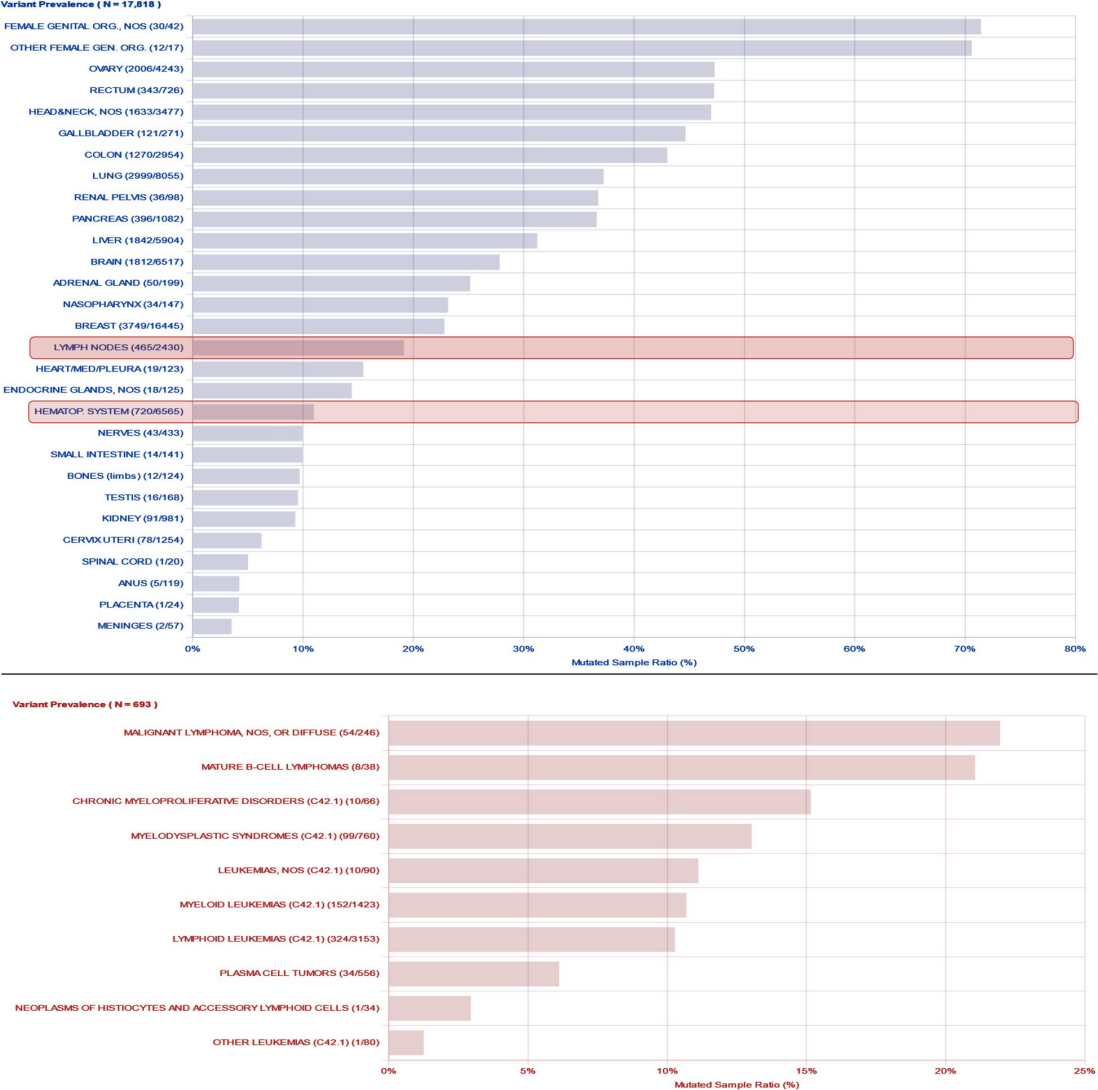
Overview of *TP53* mutation prevalence in various types of cancers, especially in hematologic malignancies. The latest database R20, July 2019 version was generated by (https://TP53.isb-cgc.org/) and modified manually

**Fig. 3:**
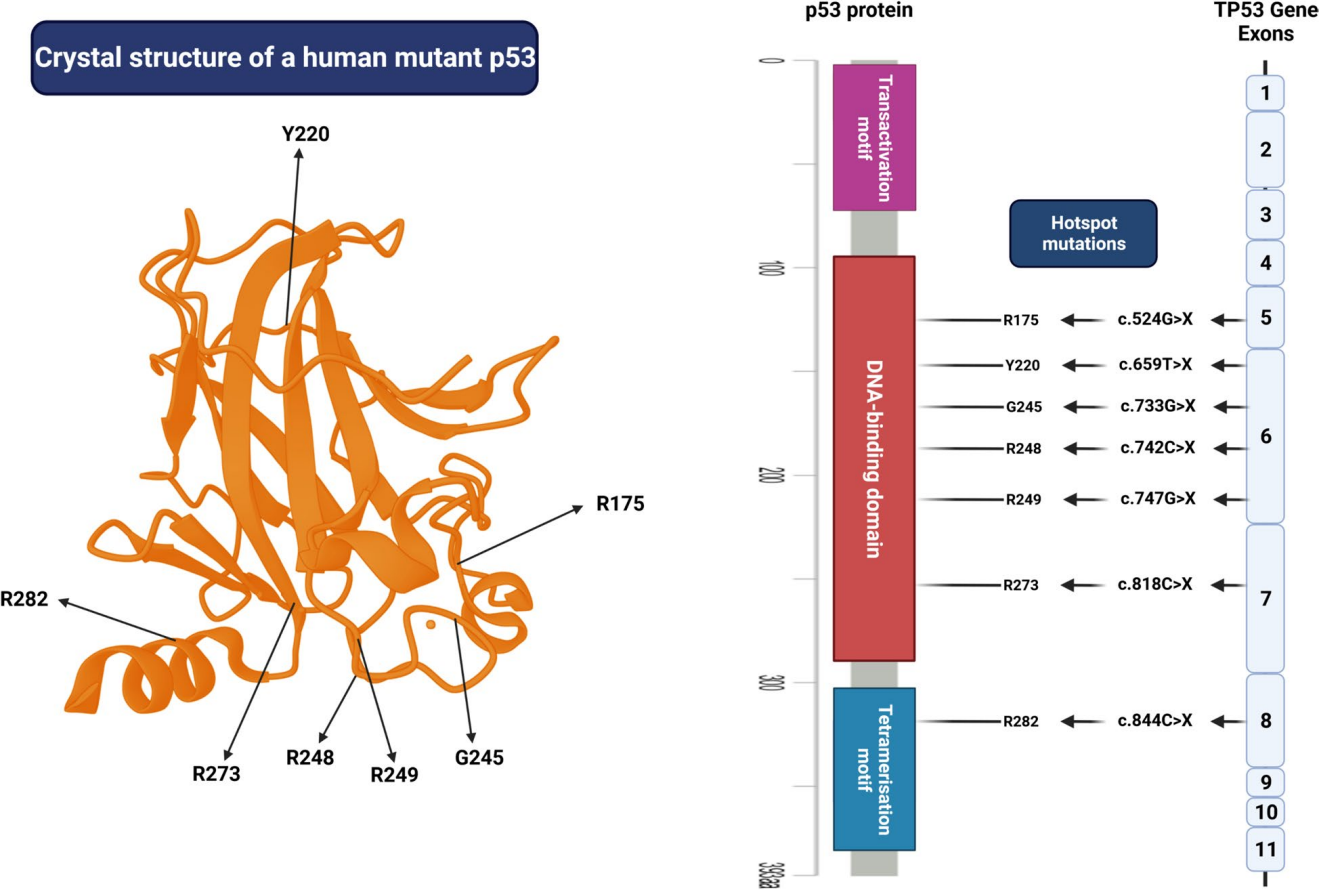
The structures and mutation hotspots of the p53 gene and protein. The crystal structure of a human mutant p53 was derived from a protein data bank (PDB ID: 4MZI)

The tumor suppressor p53, known as "the guardian of the genome," plays a critical role in maintaining genome stability. Its function can be disrupted through various mechanisms such as genomic deletion, mutation, or alterations in its regulators [109]. *TP53* aberrations are primarily found in solid tumors and are less prevalent in hematological malignancies (Fig. 2) [110, 111]. In cases where *TP53* alterations are present, there is often a strong correlation between poor clinical outcomes and resistance to therapy [112, 113]. Mutations in *TP53*, particularly in the DNA-binding domain (DBD) hotspot residues, impair its ability to bind target DNA sequences and activate gene transcription. The structures and mutation hotspots of the p53 gene and protein that disrupt the ability of p53 to bind to its target DNA sequences are shown in Fig. 3 [114–117]. While *TP53* mutations typically abrogated its tumor-suppressor function, gain-of-function mutations can provide survival advantages and resistance to chemotherapeutic drugs. These alterations are often monoallelic, with subsequent loss of heterozygosity leading to complete inactivation [115, 118]. Deletions in chromosome 17, where *TP53* is located, can also contribute to p53 inactivation, often in conjunction with mutations in the second allele. Analysis of The Cancer Genome Atlas (TCGA) dataset reveals that approximately 91% of cancers exhibit biallelic inactivation of the *TP53* gene across various tumor types [119].

High-throughput genomic technologies have identified numerous single nucleotide polymorphisms (SNPs) in *TP53*, with the most common one at codon 72 (rs1042522; P72R) encoding proline (Pro72; CC) or arginine (Arg72; GG) [120]. The arginine allele is better at inducing cellular death, while the proline allele is more efficient in activating cell cycle arrest, protecting against chemotherapy-induced apoptosis [121, 122]. However, the effects of these polymorphisms can be subtle and dependent on genetic backgrounds. Studies underscore the crucial role of p53 in tumor suppression by promoting apoptosis and cell-cycle arrest through the upregulation of genes like *CDKN1A, BAX, PUMA*, and *NOXA*. Additionally, p53 acts as a tumor suppressor by enhancing metabolic and redox balance genes such as *TIGAR, FDXR, PRKAB1,* and *SENS1* [123, 124].

Based on the IARC database (R20), *TP53* is the most common mutated gene in human cancers, nearly mutated in 50% of cancer patients. Among these, colorectal, esophageal, lung, and pancreas cancers, as well as head and neck squamous cell carcinoma, and female genital organs-related cancers like ovarian serous carcinoma, are the ones with the highest rate of *TP53* mutation [125, 126]. The majority of *TP53* somatic mutations occur in the DNA-binding core domain, with few cases reported in other p53 domains (TAD and CAD) [125]. A recent analysis of 3,281 patients across 12 major cancer types revealed a 42% *TP53* mutation rate, ranging from 2.2% in renal clear cell carcinoma to 95% in high-grade serous ovarian cancer. Studies suggest that cases with a mutated *TP53* gene and intact wild-type allele often exhibit impairment in other p53 pathway factors. These mutations in *TP53* can lead to gain-of-function (GOF), loss-of-function (LOF), and dominant-negative effects on p53 activity [127].

Particular *TP53* missense mutations that bestow p53 GOF activity differ from those causing LOF. The GOF mutations are generally known as hotspot mutations, which have a higher occurrence rate than LOF mutations [128, 129]. Contrary to LOF mutations that strip the tumor-suppressor function of p53, GOF mutations can turn p53 into an oncogene capable of disrupting cell-cycle control, modulating metabolism, inducing genomic instability, heightening differentiation, forming cancer stem cells, facilitating metastasis, and developing resistance to therapy [127, 130, 131]. Studies comparing *TP53*-null mice to mice with *TP53* missense mutations, resulting in gain-of-function (GOF) activity, show that mutant p53 can inhibit the functions of other p53 family members, p63 and p73. The oncogenic nature of p53's GOF activity suggests that missense mutations may favor tumor cell survival over complete loss of p53 function. [132, 133]. Approximately 75% of *TP53* mutations are missense, while around 10% are nonsense mutations, leading to truncated p53 proteins with impaired function. Hotspot missense mutations in p53 are prevalent in specific regions such as R175(X), R248(X), R249(X), R273(X), R282(X), and G245(X) (Fig. 3) [7, 134].

Generally, *TP53* mutations can create two types of mutant p53 known as DNA-contact mutants (R248Q-R248W-R249S-R273H-R273C-R280K-R282W) and structural mutants (R196*-R175H-Y220C- R245-R249S-R282W [135, 136]. The classification of mutant p53 types has provided different therapeutic strategies. For instance, zinc metallochaperones retrieve p53 structural form in mutants such as R175H and R273H, whereas it is ineffective against R280K mutant [137]. However, it is important to note that high levels of mutant p53 expression, such as R248Q or R280K, do not necessarily indicate high p53 activity. For example, the R248Q mutation may result in elevated protein levels, but this does not imply that the protein retains normal tumor suppressor functions. The functionality of the detected p53 protein, especially in the case of GOF mutations, remains uncertain and should be evaluated carefully when interpreting therapeutic strategies targeting p53 mutants. Further details on all mutant p53 types can be found in a detailed summary provided in Table 3.

**Table 3:** Overview of the interlaced network of mutant types of p53

Mechanism of action	Mutant type	Targets	Targeted Genes and Proteins	REF
**Metabolic reprogramming**	R175H	Protein	AMPKα/RhoA/SREBPs/ GAPDH/PGC-1α/NRF2	[138–145]
		Gene	SLC25A1/SLC7A11/ACP6/Nucleotide Metabolism Genes (NMGs)/Proteasome subunit genes	[144, 146–151]
	R273H	Protein	SREBPs/GAPDH/PGC-1α/NRF2	[141, 145, 150, 152–154]
		Gene	SLC7A11/ HMOX-1/NQO-1/Mevalonate pathway enzymes genes/ACP6/CDKN1A/NMGs/Proteasome subunit genes	[144, 148, 149, 155–161]
	G245C/P151S/R282W	Protein	AMPKα	[138]
	R280K	Protein	RhoA/NRF2	[162, 163]
		Gene	NMGs/Proteasome subunit genes/TXN	[143, 148]
	L194F	Protein	RhoA	[164]
	M237I	Protein	RhoA/ NRF2	[164, 165]
		Gene	Proteasome subunit genes	[166]
	R248Q	Protein	NRF2	[167]
		Gene	CDKN1A	[160]
	R249S	Protein	NRF2	[168, 169]
		Gene	ACP6/NMGs/Proteasome subunit genes	[144, 170, 171]
	R248W	Protein	NRF2	[172]
	G281D/G245A	Gene	SLC25A1	[127, 151, 173]
	G266E	Gene	SLC7A11	[174, 175]
	C277F	Protein	NRF2	[155, 176]
		Gene	SLC7A11	[155, 176]
	R273L	Gene	NGMs	[177]
**Survival/Chemoresistance**	R175H	Protein	P73/VDR/ETS2/NRF2/Caspase3/Caspase9/PELP1	[40, 143, 178–184]
		Gene	SLC25A1/miR-223/STMN1/REGγ/KLF17/NF-κB2/Bcl-xl/miR-128–2/MDR1/ATF3/MEF2D/dUTPase	[151, 185–195]
	R273H	Protein	NF-Y/ZEB1/p300/p73/PELP1/Caspase3	[180, 183, 184, 196, 197]
		Gene	EFNB2/SLC25A1/miR-223/STMN1/ REGγ/KLF17/NF-κB2/Bcl-xL/MDR1/dUTPase	[127, 185, 187, 189, 194–196]
	G245A	Gene	SLC25A1	[151]
	R280K	Protein	VDR/NRF2	[143, 181]
		Gene	SLC25A1/ miR-223/STMN1/ KLF17	[151, 185, 187, 191]
	D281G	Protein	ETS1	[40]
		Gene	SLC25A1/ NF-κB2/MDR1/ATF3	[151, 189, 190]
	R248W	Protein	p73/ETS2	[180, 186]
		Gene	MDR1/ATF3/dUTPase/CD95-Fas	[186, 189, 190, 195, 198]
	R273C	Protein	p73	[180]
	R280K	Protein	VDR/NRF2/ PELP1	[143, 181, 183]
		Gene	SLC25A1/miR-223/STMN1/KLF17	[151, 185, 187, 191]
	D42Y/R337H	Protein	Caspase9	[182]
	R282W	Gene	REGγ/KLF17	[127, 187, 199]
	V143A	Gene	MDR1	[186, 189]
	H179G	Gene	ATF3	[190]
**Inflammatory micro-environment**	R175H	Protein	NF-κB/DAB2IP/BTG2	[192, 200–202]
		Gene	sIL-1Rα/SDF1-CXCL12/CXC chemokines/TGFβ-R2/SOCS1/miR-1246	[203–209]
	R273H	Protein	NF-κB/DAB2IP/c-MAF	[192, 200, 201, 210]
		Gene	sIL-1Rα/SDF1-CXCL12/CXC chemokines/MMPs/IL-1 / miR-1246	[203–206, 208, 211]
	R248Q	Protein	NF-κB	[192, 200]
		Gene	CXC chemokines/MMPs/IL-1 /SOCS1	[204, 206, 209, 211, 212]
	R248W	Protein	DAB2IP	[201]
		Gene	TGFβ-R2/miR-1246	[207, 208]
	M237I/R280K	Protein	DAB2IP	[201]
	H179R	Protein	BTG2	[202]
		Gene	TGFβ-R2	[207]
	R248L	Gene	CCL2	[213]
	R172H	Gene	SDF1-CXCL12	[205]
	H179L/D281G	Gene	CXC chemokines	[206]
	V157F/R249S	Gene	miR-1246	[208]
**Invasion and metastasis**	R175H	Protein	p63/p73/SMAD3/SREBPs/Pontin/RCP/MET/Integrins/ATF3/MDM2/SP1/NRD1/Pin1/STAT3/ NF-Y	[152, 191, 214–225]
		Gene	MYO10/STMN1/PDGFR-β/NMGs/EFNB2/Twist1	[148, 196, 226–228]
	R273H	Protein	p63/p73/PTEN/SMAD3/SREBPs/Pontin/RCP/MET/Integrins/ATF3/MDM2/NRD1/STAT3/NF-Y	[152, 191, 194, 215–221, 223, 225, 229]
		Gene	MYO10/PDGFR-β/NMGs/DICER1/EFN2/c-Myc/Twist1/DLX2/NRP2/ENTPD5/let-7i	[148, 194, 224, 226–228, 230–232]
	R248W	Protein	p73/SMAD3/RCP/MET/SP1/STAT3	[191, 215–217, 225]
		Gene	REGγ/PDGFR-β/EFNB2/FOXM1/ENTPD5/let-7i	[196, 224, 227, 230, 233, 234]
	V143A	Protein	p63/p73/SMAD3/ATF3	[127, 215, 216, 219, 235]
	H179Y	Protein	p63/p73	[215]
	Y220C	Protein	p63/p73	[215]
		Gene	PDGFR-β	[227]
	I254R	Protein	p63/p73/MDM2	[220]
	R280K	Protein	p63/p73/KLF17/SP1/Pin1/	[187, 215, 222, 224]
		Gene	REGγ/KLF17/MYO10/PDGFR-β/NMGs/DICER1/EFNB2/ENTPD5/let-7i	[148, 187, 196, 224, 226, 227, 230, 232, 233]
	R175P	Protein	p73	[236]
	C135Y	Protein	SMAD3	[127]
	R282W	Protein	SMAD3/KLF17/STAT3	[187, 216, 225]
		Gene	KLF17/miR-155	[187, 237]
	R248Q	Protein	Pontin/STAT3	[191, 225]
		Gene	REGγ/miR-155/Notch3/CCNG1	[233, 237, 238]
	R249S	Protein	ATF3	[219]
		Gene	NMGs/miR-155	[148, 237]
	S241F	Gene	let-7i	[230]
	C242R/T155P	Gene	PDGFR-β	[227]
	C238F/G245D	Gene	FOXM1	[234]
	P223L/V274F	Gene	Twist1	[228]
	C176S	Gene	ENTPD5	[224]
**Proliferation enhancer/Cell-cycle destabilizer**	R175H	Protein	NF-Y/P300/TopBP1/YAP/TEAD/ETS2/STAT3	[199, 214, 239–243]
		Gene	REGγ/ NMGs/Axl receptor tyrosine kinase/cyclin A/cyclin B2/CDK1/ circPVT1	[148, 199, 241, 244–246]
	R273H	Protein	NF-Y/P300/TopBP1/MCM4/PCNA/YAP/EGFR/ETS2/STAT3/	[199, 214, 239–243, 247, 248]
		Gene	REGγ/NMGs/Axl receptor tyrosine kinase/Cyclin A/cyclin B2/CDK1/circPVT1/miR-27a/Chromatin regulators (MLL1/MLL2/MOZ)	[148, 199, 241, 244–247, 249]
	R280K	Protein	NF-Y	[239]
		Gene	NMGs/Cyclin A/cyclin B2/CDK1	[148, 241]
	V143A	Protein	TopBP1	[242]
	R248W	Protein	TopBP1/ETS2/STAT3	[214, 242, 243]
		Gene	REGγ/Chromatin regulators (MLL1/MLL2/MOZ)	[199, 249]
	R249S	Protein	TopBP1	[242]
		Gene	NMGs/Chromatin regulators (MLL1/MLL2/MOZ)	[148, 249]
	H193L	Protein	YAP/TEAD	[241, 245]
		Gene	Cyclin A/cyclin B2/CDK1/CircPVT1	[241, 244, 245]
	L194F	Protein	YAP	[241]
	R248Q	Protein	STAT3/ETS2	[214, 243]
		Gene	Chromatin regulators (MLL1/MLL2/MOZ)	[249]
	R282W	Protein	STAT3	[214]
		Gene	REGγ	[199]
	R267P/R273C/D281G	Gene	Axl receptor tyrosine kinase	[246]
	R248L	Protein	Cyclin A/cyclin B2/CDK1	[241, 244]
**Genomic Instability**	R175H	Protein	E2F4	[250]
		Gene	BRCA1/RAD17	[250]
	R273H/R248W	Protein	Mre11	[251]
**Self-Renewal/Stemness**	R175H	Gene	ALDH1A1	[252]
	R273H	Gene	lnc273–31/lnc273–34/CD44/Lgr5/ALDH1A1	[252, 253]
	R248W	Gene	CD44	[252]
	R172H	Gene	Lgr5/ FOXH1	[252, 254]

### Diagnostic methods for detecting p53 and *TP53* mutations

Studies have shown that aberrations in the *TP53* gene, such as a lack of the gene or the presence of a mutated form of it, can significantly increase the risk of developing hematologic malignancies such as leukemia and lymphoma. Determining the status of the *TP53* gene is essential for diagnosing these conditions and predicting their outcomes [255]. A variety of diagnostic techniques are employed to identify *TP53* and p53 abnormalities. Several DNA sequencing techniques are used to identify TP53 and p53 mutations, each with strengths and limitations (Table 4). These include single-stranded conformational polymorphism (SSCP) [256], denaturing gradient gel electrophoresis (DGGE) [257], capillary denaturing gel electrophoresis (CDGE) [258], and denaturing high-performance liquid chromatography (DHPLC) [259]. Each of these methods offers different advantages in detecting DNA mutations and genetic variations associated with hematologic malignancies.

**Table 4:** Diagnostic methods to detect the *TP53* mutation in various types of cancers. Latest database R20 (https://TP53.isb-cgc.org/)

Hematologic Malignancy	Diagnostic Method	Total cases	*TP53* mutation
Acute leukemia with other defined genetic alterations	SSCP	23	3
AML, minimal differentiation	SSCP	65	4
AML with other defined genetic alterations	SSCP	691	98
Adult T-cell leukemia/lymphoma (HTLV-1 positive)	SSCP	75	23
Anaplastic large cell lymphoma, T cell and null cell type	SSCP	55	4
B-cell chronic lymphocytic leukemia/small lymphocytic lymphoma	DGGE, SSCP, none	1324	163
Burkitt cell leukemia	SSCP	229	42
Burkitt lymphoma, NOS	SSCP	117	40
Chronic myeloid leukemia, NOS	SSCP	325	27
Essential thrombocythemia	SSCP	61	7
Follicular lymphoma, grade 3	DGGE	29	3
Follicular lymphoma, NOS	SSCP, DGGE	185	23
Hodgkin lymphoma, NOS	DGGE, SSCP	33	4
Langerhans cell histiocytosis, NOS	OncoMap	34	1
Leukemia, NOS	NIRCA	67	7
Lymphoid leukemia, NOS	SSCP	128	18
Malignant lymphoma, large B-cell, diffuse (various subtypes)	SSCP, DGGE, NGS	518	111
Malignant lymphoma, non-Hodgkin, NOS	SSCP, DGGE, CDGE	441	89

Non-Invasive Genomic Risk Assessment (NIRCA) [260] and Functional Analysis of Separated Alleles in Yeast (FASAY) [261] are two additional techniques used in the diagnosis of *TP53* and p53 mutations in hematologic malignancies. NIRCA uses targeted deep sequencing of circulating tumor DNA [260], while FASAY, assesses the impact of *TP53* mutations on p53 protein function using yeast cells.

Fluorescence in Situ Hybridization (FISH) is routinely used to identify Del(17p), the chromosomal location of *TP53*, but this method is not always effective. Thus, it is vital to test for relevant *TP53* mutations using various methods (Table 4) [262].Other methods such as direct Sanger sequencing [263, 264] and Next-Generation Sequencing (NGS) are also employed, with NGS offering higher specificity and sensitivity [264, 265]. Moreover, Microarray resequencing provides high sensitivity but only detects mutations for which the array probes were printed [263, 266]. Immunohistochemistry (IHC) staining of p53 proteins in the nucleus can therefore be a surrogate marker for the *TP53* mutation [267]. Most studies indicate that setting the threshold for immunohistochemical evaluation of p53 at ≥ 10% is optimal. However, it's important to note that different antibodies used in IHC may have varying thresholds [268–270]. In recent years, liquid biopsy has provided a way for non-invasive detection of *TP53* mutations via circulating tumor cells (CTC) and cell-free DNA (cfDNA) [271]. The methods presented here have made it possible to detect *TP53* mutations in each hematological malignancy, which we have covered in detail further in this review.

Based on the latest database (R20) from the National Cancer Institute (https://TP53.isb-cgc.org/), Table 4 represents a comprehensive overview of *TP53* mutations across various hematologic malignancies. Of the 4,431 cases, using different diagnostic methods, 664 were found to harbor *TP53* mutations, yielding an overall mutation rate of 15.0%. Notably, B-cell chronic lymphocytic leukemia/small lymphocytic lymphoma had the highest total cases (1,324), with 163 TP53 mutations, corresponding to a mutation rate of 12.3%. Burkitt lymphoma NOS showed a notably high *TP53* mutation rate, with 40 mutations out of 117 cases (34%). Similarly, AML with other defined genetic alterations, demonstrated high heterogeneity with a mutation rate of 14.2% across 691 cases, reflecting its clinical complexity. Conversely, Langerhans cell histiocytosis NOS had a low mutation rate, with only one *TP53* mutation detected out of 34 cases (2.9%).

Furthermore, in Adult T-cell leukemia/lymphoma, the *TP53* mutation frequency was particularly high, with 23 mutations in 75 cases (30.7%), indicating a significant correlation between *TP53* mutations and poor prognosis in this group. Across the spectrum of hematologic malignancies, Malignant lymphoma, large B-cell, diffuse and non-Hodgkin lymphoma, NOS exhibited relatively high *TP53* mutation rates, reinforcing the prognostic significance of *TP53* alterations in aggressive lymphoid malignancies.

## p53 in hematological malignancies

### Acute myeloid leukemia (AML)

AML is the most common acute leukemia in adults [272], characterized by abnormal proliferation and differentiation of a clonal population of myeloid stem cells in the bone marrow and peripheral blood [273]. The 2022 WHO Classification of Acute Myeloid Leukemia (AML) provides a detailed framework to categorize AML into two major groups: AML with defining genetic abnormalities and AML defined by differentiation.

AML with Defining Genetic Abnormalities includes subtypes characterized by specific genetic alterations that play a key role in the pathogenesis and prognosis of the disease. This group encompasses acute promyelocytic leukemia (APL) with *PML::RARA* fusion, which is distinct due to its responsiveness to targeted therapy with all-trans retinoic acid (ATRA) and arsenic trioxide. Other subtypes in this category are defined by specific fusion genes, such as AML with *RUNX1::RUNX1T1* fusion, AML with *CBFB::MYH11* fusion, AML with *DEK::NUP214* fusion, AML with *RBM15::MRTFA* fusion, and AML with BCR::ABL1 fusion. Additionally, AML with *KMT2A, MECOM*, and *NUP98* rearrangements highlights subtypes with various chromosomal translocations. Subtypes also include AML with *NPM1* mutation and AML with *CEBPA* mutation, which have distinct clinical and prognostic implications. The category of AML, myelodysplasia-related (MRC) encompasses AML that arises in the context of a prior myelodysplastic syndrome or exhibits significant dysplastic features. The classification also recognizes AML with other defined genetic alterations, capturing additional, less common genetic abnormalities [274].

AML Defined by Differentiation classifies subtypes based on the extent of differentiation of the leukemic cells. This includes AML with minimal differentiation, where the blasts lack significant evidence of maturation; AML without maturation, where the blasts show some differentiation but lack complete maturation; and AML with maturation, characterized by a greater degree of differentiation among the leukemic cells. It also includes specific morphological variants such as acute basophilic leukemia, acute myelomonocytic leukemia, which shows both granulocytic and monocytic differentiation, acute monocytic leukemia with predominant monocytic features, acute erythroid leukemia, marked by a significant erythroid component, and acute megakaryoblastic leukemia, characterized by blasts that show megakaryocytic lineage differentiation [274].

The status of p53 alterations varies across different subtypes of Acute Myeloid Leukemia (AML). *TP53* mutations are generally less frequent in de novo AML, occurring in about 10% of cases but they are more common in therapy-related AML (t-AML) and secondary AML (s-AML) that arises from a prior myelodysplastic syndrome (MDS). Most *TP53* mutations in AML are missense mutations located between codons 125 and 300, particularly in the DNA-binding domains encoded by exons 5–8 [275]. In terms of prognostic impact, *TP53* mutations in AML are associated with poor outcomes, including resistance to chemotherapy and shorter overall survival (OS). The mutations can be classified as "single-hit" or "multi-hit" based on their allelic status. "Single-hit" refers to the presence of a single *TP53* mutation, while "multi-hit" indicates two different mutations or a single mutation with a high variant allele frequency (VAF) (> 50%) or accompanied by a 17p loss. "Multi-hit" *TP53* mutations are significantly associated with high-risk disease features, such as complex karyotype (CK), and are linked to worse overall survival (OS) and disease-free survival compared to cases without *TP53* mutations or with single-hit mutations [275].

For specific AML subtypes, *TP53* mutations are particularly prevalent in subtypes characterized by myelodysplasia-related changes, reflecting their origin from a background of genetic instability. In contrast, subtypes defined by recurrent genetic abnormalities, such as those with PML::RARA or RUNX1::RUNX1T1 fusions, generally have fewer *TP53* mutations. The presence of *TP53* mutations in these categories contributes significantly to the heterogeneity of AML and underscores their role as a driver of poor prognosis and treatment resistance [275, 276].

It is noteworthy that alterations such as a CK (defined by at least three unrelated cytogenetic abnormalities), monosomal karyotype (MK) (defined by at least two autosomal monosomies, or one autosomal monosomy associated with at least one structural abnormality), -5 or del(5q), -7, t(6;9), inv(3) and 11q23 are associated with poor response to treatment and shorter overall survival (OS) [277].

In addition to chromosomal rearrangements, molecular alterations, including the presence or absence of specific gene mutations and/or changes in gene expression, are implicated in the development of AML [278, 279]. In this regard, several studies have shown that *TP53* alterations affect AML evolution, biology, and therapy response. *TP53*-mutated AML is a subset of AML with poor response to chemotherapy (28%-42% overall response rates) [280, 281] and dismal outcomes [282]. Deletions, insertions, and nonsense mutations are detected in *TP53*; however, missense mutations are the most common type of variants (> 80%) [283].

A majority of de novo AMLs retain intact, unaltered *TP53,* and fewer than 10% of AML patients carry *TP53* mutations [284–286]. In contrast, *TP53* alterations, including loss of chromosome 17, mutations, and/or deletions in this gene, are frequent in subjects with adverse cytogenetics such as CK-AML and MK-AML [287, 288]. The incidence of *TP53* mutations in AML with complex karyotypes (CK) varies across studies, ranging from 53% in British and American cohorts to 78% in a German study. This variability is likely attributable to differences in study design, sample size, or methodologies rather than the nationalities of the patients. Regardless, *TP53* mutations consistently predict resistance to conventional chemotherapy and are associated with a poor outcome [281, 288–293]. Ohgami and colleagues further analyzed the association of individual mutations with AML subtypes and found that *TP53* mutations were notably linked to AML with high-risk cytogenetics, specifically in cases with a CK. Among 15 cases with a CK, 8 (53%) exhibited *TP53* mutations, whereas none of the 78 AML cases without a CK had this mutation. To confirm this relationship, they sequenced an additional 21 AML cases with CK, revealing that 12 cases (57%) had *TP53* mutations. Additionally, *TP53* mutations were frequently associated with chromosome 17p deletions (del(17p)); out of 20 cases with *TP53* mutations, 11 (55%) also had del(17p), while 2 cases without del(17p) contained two independent *TP53* mutations. These findings suggest that *TP53* mutations may act as a secondary event in the development of leukemia, occurring after the initial chromosomal instability associated with CK [289]. Somatic *TP53* mutations are also correlated with chromothripsis (massive chromosome rearrangements in a one-step catastrophic event) in AML patients with CK [294, 295]. Furthermore, mutational analysis of *TP53* is an important additional tool to predict outcomes after hematopoietic stem cell transplantation (HSCT), as it correlates with worse OS [296] and increased risk of relapse [297] in those patients. Among adverse cytogenetics, MK is identified as the factor with the worst prognosis in AML cases [298, 299]. Yanada et al. detected *TP53* mutation in 16% of elderly AML patients (aged 60 years or older), which was frequently accompanied by MK and adverse outcomes [280]. *TP53* mutations may occur across almost all FAB morphologic subtypes; however, there is a modest increase (25–36%) in M6-erythroleukemias [281, 300, 301]. Acute megakaryoblastic leukemia (AMKL) is a rare subtype of AML, commonly seen in children with DS. Approximately 10% of infants with DS exhibit a unique characteristic known as transient myeloproliferative disorder (TMD). Although TMD resolves spontaneously in the majority of affected infants, 20% of TMD patients develop AMKL within three years, hinting at the involvement of genetic alterations during the progression from TMD to AMKL. There is a hypothesis that *TP53* mutations might play a role in the evolution from TMD to AMKL. One study reported that 2 out of 3 AMKL patients had *TP53* mutations, but all seven TMD did not [302]. Nevertheless, Hirose et al. found no relationship between the evolution of AMKL and *TP53* mutation [303].

p53 alterations have also been analyzed in therapy-related myeloid neoplasms (t-MNs). The incidence of *TP53* mutations is higher in t-AML compared with de novo AML (21%-38% vs. less than 10% respectively) and is associated with 5q-, CK, and poor prognosis [304–307]. However, the mechanism by which *TP53* mutations are augmented in t-MNs is not clear. According to the WHO classification, t-MNs are attributable to mutations in hematopoietic stem and precursor cells (HSPCs) induced by treatments [308]. Intriguingly, prior cytotoxic treatment cannot directly induce genome-wide DNA damage or a leukemia-specific mutation but rather may facilitate the preferential expansion of a pre-leukemic clone harboring somatic *TP53* mutations, which are resistant to chemotherapy. Indeed, the mutation remains dormant for many years in HSPCs, until chemotherapy exposure [305, 309]. The presence of *TP53* germline mutations is 1.1% in primary AML cases versus 5.6% in t-AML, highlighting the importance of this genetic event in the development of t-AML [310]. *TP53* alterations were also assessed in s-AML, currently classified as AML-MRC [311]. The frequency of *TP53* mutation in this subgroup of AML varied from 22% to 27.3% in different studies [312, 313] and is associated with inferior outcomes and shorter OS [312, 314, 315]. Moreover, McGraw et al. reported an increased p53 expression in AML-MRC patients, interrelated with *TP53* mutation, higher risk of disease, and inferior OS [316].

Loss of *TP53* is found in 40%-75% of CK-AML patients [291, 317] and 53.6% of MK-AML cases [318], while only present in approximately less than 5% of normal karyotype (NK) AML cases [285, 319]. A significant positive correlation between *TP53* deletion and established high-risk aberrations, including del (5q), -5, -7 was also reported in AML [317, 320]. Previous multivariate analysis reveals that AML patients with a single *TP53* deletion have a worse treatment outcome than patients with NK, and it was suggested that *TP53* deletion should be classified into the high-risk category for developing risk-adapted treatment strategies in AML [320]. Accordingly, in the latest classification, AML with *TP53* deletion is included as a subgroup of myeloid neoplasms following cytotoxic therapy (MN-pCT), recognizing the poor prognosis associated with this genetic alteration. The majority of cases of MN-pCT are linked to *TP53* mutations, which are particularly detrimental when present as biallelic or "multi-hit" alterations, defined as the presence of two or more *TP53* mutations or a single *TP53* mutation accompanied by a 17p deletion or copy-neutral loss of heterozygosity (cnLOH) at the *TP53* locus. These biallelic *TP53* alterations are associated with highly adverse outcomes. In contrast, less frequent mutations involve genes such as PPM1D and other DNA damage response genes, which may warrant further investigation for potential germline predisposition, indicating a need for additional genetic work-up in these patients [274].

A great number of studies examined the impact of SNP in codon Arg72Pro of p53 on cancer susceptibility and therapy outcome. There is no association between AML risk and p53 codon 72 polymorphism in Indian [321], Chinese [322], American [323], Egyptian [324, 325], Caucasians from Germany, Austria [326], and Japanese [327] populations. Likewise, two meta-analyses of 6 [328] and 14 case–control studies [329] revealed no significant correlation between p53 codon 72 polymorphism and AML susceptibility. *TP53* codon 72 polymorphism alone is not associated with altered risk of AML, while the coinheritance of the G allele of MDM2 SNP309 cooperates to increase disease risk [330]. However, Bezerra et al. study showed that the *TP53* Pro/Pro genotype was correlated with a higher risk of leukemia and favorable outcome in Brazilian AML patients who received conventional therapy, especially those with intermediate cytogenetic risk [331]. It is important to note that the difference concerning the risk of AML is attributable to the Brazilian genetic background of the study, which is well-known for its mixed genetic population. Moreover, a sizeable proportion of patients were excluded from the survival analysis which could bias the analysis. The median follow-up was only 135 days, masking late events [332]. The relationship among p53 codon 72 polymorphism, clinical and laboratory features, as well as treatment outcomes, are also investigated in AML patients. There is a possible trend toward worse OS among AML patients with SNP P72R [333]. Abdel Hamid et al. reported that patients with homozygous Arg/Arg at codon 72 of p53 had better median OS months compared with Arg/Pro and Pro/Pro and concluded that p53 was an independent prognostic factor of survival [334]. In another investigation, no significant correlation was identified between p53 codon 72 polymorphism and clinical parameters (gender, cytogenetic risk, FAB subtype, etc.) in Chinese patients with AML [322]. The result of a study conducted on Japanese patients with AML reveals that the p53 genotype at codon 72 is effective in detecting LOH, but is not related to clinical features or therapeutic response of AML [327].

Most of the *TP53* genomic alterations disrupt p53 protein homeostasis and increase its intracellular level, which is detectable by IHC [335, 336]. p53 protein levels were detected in a high percentage of AML cases. In the Assi et al. study, 24% of the de novo AML patients with normal diploid cytogenetic, regarded as an intermediate-risk category [334], had p53 overexpression, which was accompanied by lower platelet counts, lower frequency of CD34 expression in blasts, higher bone marrow blast counts, and a higher frequency of *FLT3* internal tandem duplication in this subset of AML patients. p53 expression was also correlated with shorter leukemia-free survival (LFS) in patients who were consolidated with allogeneic stem cell transplantation (allo-SCT). Thus, they suggested evaluating p53 expression by IHC as a potentially valuable tool that could help indicate distinct clinical characteristics of patients with normal diploid cytogenetics and identify cases who could benefit from post-SCT maintenance therapy [337, 338]. In another investigation, a high level of p53 expression was observed in AML-MRC versus AML- not otherwise specified or AML with recurrent genetic abnormalities. Additionally, higher p53 expression was correlated with *TP53* mutations, CK, and worse OS in this subset of AML [337]. Cleven et al. examined p53 expression in two separate cohorts of patients with t-AML and t-MDS. IHC-positive p53 staining was detected in 31% and 28% of patients with t-AML in both cohorts. Strong p53 immunostaining was highly predictive of a *TP53* gene mutation in t-MNs and associated with decreased OS, particularly in the subset of patients who receive SCT [336]. Increased p53 protein expression is also a potential predictor of early relapse after HSCT in children with AML [339]. These observations support the assessment of p53 expression profiling via IHC besides the mutational status of the *TP53* gene. Analyzing p53 expression may provide valuable prognostic information and could improve current AML risk stratification algorithms.

### Acute lymphoblastic leukemia (ALL)

ALL is an aggressive transformation and proliferation of lymphoid progenitors of both B and T-cell origin. It is the most common cancer in children, accounting for approximately 25% of childhood malignancies and characterized by a 90% long-term survival rate for standard-risk pediatrics [340]. Various cellular pathways are mutated in almost ALL cases, including *TP53*. Multiple investigations evaluated genetic variants in the *TP53* gene as well as their association with leukemogenesis and the outcome of therapy. Knowing the status of *TP53* and target p53 target therapies might help achieve complete remission (CR).

Hendy et al. identified *TP53* mutations in 13% of newly diagnosed children with ALL, 10.3% of those in complete remission (CR), and 17.6% of the relapsed group, demonstrating a significant difference between these groups [341]. In another study, *TP53* mutation, and as a result, its overexpression were not common events in a group of 62 children with de novo ALL (14.5%), and alone, it could not be considered as strong independent predictors of outcome; however, p53 alterations were associated with the resistance to the induction therapy with prednisone [342]. *TP53* deletion is identified in 9% of de novo B-ALL cases and 18% of T-ALL cases [343]. In a cohort of 3,801 children with newly diagnosed B-cell ALL, 49 unique non-silent rare *TP53* coding variants were identified in 77 cases (2.0%), with 22 of these variants classified as pathogenic. In contrast, *TP53* alterations were more prevalent in pediatric ALL patients with relapse. Yu et al. reported *TP53* mutation incidences of 29% in childhood relapsed B-cell ALL and 46% in relapsed T-cell ALL. Notably, nearly 70% of all TP53 mutations and deletions were relapse-specific. This highlights their potential role in disease recurrence [344]. Moreover, *TP53* was altered in 12.4% and 6.4% of first-relapse childhood ALL patients with B-cell precursor ALL and T-cell ALL, respectively, with approximately half of the *TP53* alterations gained at relapse [345]. Analyzing exon 5 in the *TP53* gene in relapsed childhood ALL revealed a mutation in 19% of patients (12% in B-lineage and 33% in T-lineage), most of them were identified in the relapse phase. In two exceptional subjects, one mutation was observed as a germline mutation and one was already present at the diagnosis [346]. Hof et al. reported p53 alterations in 6 out of 81 (7.4%) children with the first relapse of T-cell ALL, of which five cases with *TP53* alterations presented complete loss of the wild-type allele [347]. Notwithstanding, Gump et al. reported no *TP53* mutation in 17 relapsed childhood ALL patients, probably due to relatively small sample numbers [348]. Overall, *TP53* mutations and deletions occur more frequently in pediatric B-ALL patients with relapse versus primary and correlate with inferior outcomes [349].

Salmoiraghi et al. reported *TP53* mutations in 14 of 171 (8%) adult Philadelphia-negative ALL cases, interrelated with worse OS and higher cumulative incidence of relapse (CIR) [350]. A similar result was obtained from Chiaretti et al. study in which *TP53* mutation occurred in 8.2% of adult ALL cases at diagnosis [351]. A lower incidence of *TP53* mutation (4.5%) was also identified by whole-genome genotyping assay in younger adult ALL patients [352]. Kanagal-Shamanna et al. reported *TP53* mutation in approximately 15% of newly diagnosed adult ALL patients with a significant association with low-hypodiploid karyotype but not with survival and CR duration after frontline treatment with HCVAD-based regimens. The finding that *TP53* mutation was not associated with adverse outcomes might be attributable to receiving a monoclonal antibody as part of the induction and consolidation regimen in about 90% of patients. Hence, chemoimmunotherapy might overcome the poor prognostic effect of *TP53* mutation in ALL patients [352]. In a cohort including a mixture of adults (mostly), a relatively low proportion of childhood, and Burkitt/MYC + ALL, the frequency of *TP53* mutation was 15.7% and occurred together with a low hypodiploid karyotype and MYC-translocations. Patients with *TP53* mutation had shorter survival, especially when accompanied by wild-type *TP53* allele loss [353]. Notably, experiments on a mixed group of children and adults diagnosed with B-ALL showed higher *TP53* mutation frequency in adults than children (20.2% vs. 7.6% respectively) [354].

Almost 75% of ALL patients carry acquired chromosome abnormalities which can be divided into two major groups: (1) an alteration in the number of chromosomes (ploidy), (2) the presence of translocations that generate leukemia-specific fusion transcripts or deregulate the gene expression [355]. Chromosome number or ploidy is an important indicator of prognosis and risk stratification in patients with B-ALL, especially those without translocations. Hyperploidy with a chromosome number greater than 50, found in approximately 25% of children with ALL, confers a favorable clinical outcome [356]. In contrast, hypodiploid acute ALL with chromosome numbers less than 44 conjoin with a poor prognosis and comprises up to 5% of childhood ALL patients [357, 358]. Hypodiploid ALL have been subclassified into: (1) high hypodiploidy with 40–43 chromosomes, (2) low hypodiploidy (LH) with 32–39 chromosomes, and (3) near-haploidy (NH) with 24–31 chromosomes [359]. Doubling of either a low-hypodiploid or a near-haploid clone results in a high-hyper diploid karyotype, referred to as masked low hypodiploidy (mLH) and masked near-haploidy (mNH), which have an extremely poor prognosis in ALL cases, often misclassified for risk [360]. Interestingly, LH-ALL is highly enriched for alterations in *TP53*. Holmfeldt et al. reported somatic *TP53* mutations in 91.2% of the childhood LH-ALL samples, which majority of them were homozygous due to the loss of the wild-type *TP53* allele. Almost half of the studied cases exhibited the same *TP53* mutations in non-tumor cells, indicating that these were germline alterations. They also found somatic *TP53* mutations in 90.9% of adults with LH-ALL, with no evidence of a germline *TP53* mutation [361]. In another investigation, 19% of children with hypodiploid ALL carry germline *TP53* mutations [362]. A high frequency of somatic *TP53* mutations was observed in 93% of ALL cases with LH karyotype, which in most of them, the other *TP53* allele was lost due to the monosomy 17 [355]. In Fang et al. study, the results of chromosome genomic array testing (CGAT) analysis revealed a unique case of adult B-ALL with mLH and a somatic *TP53* mutation. Moreover, they identified three cases of pediatric B-ALL with mLH, two with *TP53* mutations and one untested [360]. These observations confirm alterations of *TP53* as a hallmark of hypodiploid ALL, distinguishing this rare subgroup from other ALL patients. It also implies that adverse outcomes of this particular ALL subset might be due to high *TP53* mutation frequency.

There is a linkage between the incidence of *TP53* mutations and the type of chromosomal translocations in B-precursor ALL cell lines [363, 364]. Furthermore, a study revealed that *TP53* mutation was more frequent among MLL rearranged and t(1;19)-positive ALL cell lines than Philadelphia chromosome-positive and t(17;19)-positive ALL cell lines [365]. Few studies have investigated germline mutations in ALL. Carrying germline *TP53* pathogenic variants correlates with ALL leukemogenesis, inferior survival and higher risk of second cancers [366]. As mentioned earlier, *TP53* germline mutations are a feature of the LH phenotype in pediatrics with ALL [361, 362]. Some studies suggested that *TP53* mutations did not trigger familial ALL [367, 368]. A review of a large cohort of *TP53* mutation-positive extended kindreds demonstrated only one family with multiple cases of ALL, suggesting that familial ALL is not usually correlated with *TP53* mutations [369]. However, Powell et al. performed Whole-exome sequencing in an extended Hispanic kindred and found *TP53* as an ALL susceptibility gene [370].

Several studies also described an association between *TP53* exon 4 polymorphism Arg72Pro and increased risk of ALL development [371, 372]. An augmented risk of adult ALL accompanied by the p53 Pro/Pro genotype in a Chinese population [373]. Moreover, the result of Cabezas et al. study on 173 Caucasian ALL cases revealed that patients harboring *TP53* Pro/Pro or Pro/Arg genotype had a higher risk of resistance to apoptotic-based agents [373]. However, p53 codon 72 polymorphisms did not strengthen the risk of ALL development in an Indian population [374].

Not much information is available about the methylation of the *TP53* in ALL. Hypermethylation of the promoter regions of tumor suppressor genes correlates with transcriptional silencing and disease progression [375]. In a cohort of 57 ALL patients, the incidence of *TP53* mutation, one *TP53* allele deletion, and promoter hypermethylation was 8.8%, 7.5%, and 32% respectively [11]. In another similar short report, a high proportion of ALL samples (32%) showed *TP53* promoter methylation at diagnosis, correlated with decreased gene expression and functional impairment of this tumor suppressor gene in ALL [376]. These observations suggest that methylation of the *TP53* promoter is a more frequent molecular event in ALL compared to mutation and deletion and support the concept that methylation could represent a mechanism for *TP53* inactivation in ALL.

Overexpression or dysregulation of some oncogenes may have a critical role in leukemogenesis by affecting the regulation of intracellular growth or suppressing apoptosis [377]. Aberrant expression of p53 was reported in several studies [377, 378]. Alteration in p53 function is more frequent in ALL children with early treatment failure compared to cases who remained in long-term continuous remission [379]. In a recent investigation, p53 mRNA levels of 146 children with ALL as well as 23 child donors with idiopathic thrombocytopenic purpura (ITP), were examined. p53 mRNA level was higher in patients with ALL in comparison with that in the ITP donors, and increased p53 expression was correlated with poor OS and relapse-free survival (RFS) in childhood ALL [380]. Mattsson et al. reported an elevated p53 expression in the bone marrow of pediatric ALL patients at 0‐3 months after HSCT, associated with an increased risk of relapse. They suggested the prognostic role of p53 for predicting relapse in pediatric ALL patients who undergo HSCT [381]. In another study, immunohistochemical expression of p53 was evaluated in bone marrow samples from two groups at the time of diagnosis: (1) 30 children with ALL who survived disease-free for at least 5 years and (2) 15 advanced ALL patients admitted for bone marrow transplant (BMT). p53 was overexpressed in 4 out of 30 ALL patients in the relapse-free group. At the same time, 8 out of 15 advanced ALL cases overexpressed p53 before BMT, implying that p53 might be overexpressed in more aggressive leukemias [382].

In a recent report by Chitadze and colleagues [383] they analyzed *TP53* mutations in 43 adult ALL patients, revealing their persistence even during molecular remission. While 70% of patients were MRD-negative, 30% still harbored *TP53* mutations, suggesting these mutations originate in preleukemic progenitor cells. This challenges the use of standard MRD assessments, as *TP53* mutations can persist in non-leukemic cells, complicating the interpretation of remission. The study also highlighted the role of *TP53* mutations in relapse and disease progression. A case of relapse in one patient showed clonal evolution with the retention of the original *TP53* mutation, alongside a new mutation, suggesting clonal heterogeneity. This underlines the potential of *TP53* mutations to drive treatment resistance and relapse. Chitadze et al.’s findings emphasize the need for careful consideration of *TP53* mutations in MRD monitoring and treatment, as these mutations may serve as early leukemogenic events that contribute to disease persistence and relapse [383]. Further research is needed to fully understand their clinical implications and to guide treatment strategies in ALL.

### Chronic myeloid leukemia (CML)

CML is a myeloproliferative neoplasm that contains roughly 15% of newly diagnosed cases of leukemia in adults [384]. The cornerstone of CML pathogenesis is the presence of the Philadelphia chromosome, which arises from the fusion of the Abelson oncogene (ABL) from chromosome 9 with the breakpoint cluster region (BCR) gene on chromosome 22. This results in the expression of an oncoprotein called BCR‐ABL1 [385]. CML can be classified into three phases: chronic phase (CP), accelerated phase (AP), and blast phase (BP), also known as the acute phase or blast crisis (BC). CML frequently presents in CP, characterized by the clonal expansion of mature myeloid cells and the specific response of leukemia cells to normal regulators [385, 386]. Untreated CML patients will eventually evolve to a lethal BP, distinguished by unrestrained proliferation without differentiation and loss of response to normal control mechanisms [387]. This phase of CML typically resembles acute leukemia (myeloid in 60%, lymphoid in 30%, megakaryocytic or undifferentiated in 10%) [388]. Most patients progress into AP before BP with warning signals such as worsening anemia, splenomegaly, and organ infiltration. Nonetheless, 20% of patients progress into BP without entry into the AP phase [388].

The heterogeneity of BC types may be due to the different molecular and chromosomal alterations observed in this stage of the disease. The acquisition of more mutations, but not changes in the chimeric BCR-ABL gene, may lead to BP [389, 390]. The transition from CP to BC is accompanied by a spectrum of alterations in p53 gene expression followed by changes in gene structure such as rearrangement, deletion, or point mutation [391, 392]. The p53 inactivation often results from point mutations that involve one allele with or without loss of the other allele. There are various inconsistencies in the incidence of *TP53* mutations in CML. However, most studies confirm the evolution of the disease to BC with an increasing incidence of *TP53* mutations [392, 393]. *TP53* mutations are rare at the time of diagnosis and the CP phase of CML [394, 395]; however, they were present in approximately 15%-30% of the CML patients in BC [396]. The *TP53* mutations are concurrently associated with the loss of the short arms of chromosome 17 (17p), mainly through the formation of an isochromosome 17q [i(17q)]. The i(17q) chromosome occurs in approximately 30% of patients in BC, [397] and almost within 40% of these cases, p53 mutations existed on the remaining p53 allele [392]. This hints at the possibility that loss of p53 on chromosome 17pl3, besides *TP53* point mutations, may result in the inactivation of both normal p53 alleles and the progression of CML to the AP. The result of a survey from 13 CML patients revealed 8 BC cases with a significant loss of allele (LOA) rate for the p53 gene and also *TP53* mutation in 2 of these 8 cases. Furthermore, p53 rearrangements were detected in six patients with myeloid BC and two cases with an erythroid and an undifferentiated BC. However, patients with lymphoid BC had no evidence of p53 rearrangements, suggesting an increased LOA rate was linked to myeloid BC [394]. Moreover, Feinstein et al. demonstrated that in around 25% of CML patients in the AP, one p53 allele was lost -e.g., by the i(17q) aberration- which was associated with the inactivation of the other allele through the loss of expression, rearrangement, or point mutation. In contrast, in an analysis of some patients who carried both p53 alleles no p53 alterations were indicated. Thus, they concluded that p53 mutation with a loss of function was the undelaying reason for the progression of about 25% of CML patients.[398]. Nakai et al. reported p53 gene mutation in 5 out of 31 BC patients and in 1 out of 7 cases in AP. Among those six samples with p53 gene mutations, loss of a 17p was detected in five of them, and only one lymphoid patient had intact normal chromosome 17 homologous. These observations suggest that mutations of the p53 gene were closely associated with the myeloid crisis with loss of a 17p. Their result also divulged that in 4 cases with the loss of a 17p and p53 mutation, survival periods after blastic transformation were shorter than those with the loss of a 17p but without p53 mutation. Hence, the leukemic clone might become more aggressive when the p53 gene is altered on the residual 17p.[392]. Consistent with the proposal that alterations in the structure of the p53 gene might have a role in the evolution of the disease, Ahuja et al. observed deletions and rearrangements of p53 in 8 of 34 patients in BC and 1 of 4 patients in the AP, but in only 1 of 38 patients in the CP of CML [393].

Conversely, almost half of the cases with a loss of 17p do not exhibit *TP53* inactivation. Gaidano et al. reported that p53 was not frequently involved in the molecular pathogenesis of BC because p53 mutations were restricted to only one case of 26 tested. The loss of 17p in the absence of p53 mutations was also observed in 2 patients with myeloid BC out of 26 tested cases, suggesting that a tumor-suppressor gene other than p53 might be involved in tumors exhibiting the 17p- abnormality [399]. In another investigation, the alteration of the p53 was investigated in (17q)-positive CML patients, and no structural abnormalities of the remaining p53 allele were detected [400]. Similarly, the search for mutations inactivating the p53 tumor suppressor gene revealed that this event was rare in CML-BC, as one out of twenty (5%) CML-BC patients harbored p53 mutation [401]. The result of a study performed on three CML patients with 17p aberrations at different stages of disease (CP, AP, and BC) demonstrated only one case with del (17) in BC with p53 expression. Moreover, single-strand conformation polymorphism (SSCP) analysis indicated this expression due to a *TP53* mutation. The two other patients with normal PCR/SSCP analysis for *TP53* mutation and negative p53 expression, progress to lymphoid BC. These findings indicate the cytogenetic alterations in 17p are independent of mutations in the *TP53* gene [402].

Reports regarding gene/protein expression of p53 were conflicting. It is shown that p53 is overexpressed in several leukemic cell lines as well as the abnormal cells from patients with different hematologic malignancies. In contrast, a negligible level of p53 protein is expressed in normal counterparts [403, 404]. The result of an investigation on CML cell lines revealed that none of them expressed detectable p53 protein, while accumulated p53 transcripts were found in EM-2 and EM-3 cell lines. Myeloid cells of 3 of 4 CML patients also overexpressed p53 mRNA, proposing that expression of the gene is not regulated normally in CML [404]. Cavalcanti et al. stated that 12 out of 41 (29.2%) CML patients overexpressed p53 protein (mostly seen in the AP and BC of the disease), accompanied by multidrug resistance (MDR) function [405]. However, Iolascon et al. reported that p53 gene expression was not changed in either chronic or acute phases of CML [406]. A normal p53 gene expression, which encodes an intact amino acid sequence, was also observed in the majority of patients in BC [398]. These observations suggest that alteration in other cell cycle regulatory genes might contribute to the fatal BC.

Some researchers have addressed the influence of germline polymorphism at codon 72 *TP53* on CML susceptibility and clinical response to tyrosine kinase inhibitors, e.g., imatinib. Weich et al. found that *TP53* c.213 G > C (Arg72Pro) polymorphism might be involved in CML development in an Argentinean population. They also demonstrated that patients carrying the *TP53* -GG (Arg/Arg) genotype had higher levels of BCR-ABL1 transcripts and were more likely to have a worse clinical prognosis compared to those cases with other genotypes (GC/CC) [407]. Likewise, a significant association is between *TP53* -GG (Arg/Arg) genotype and high Sokal score as well as failure to imatinib treatment [408]. In discordance with previous studies, Bergamaschi et al. reported a significant correlation between *TP53* -C (Pro) allele and poor cytogenetic response in 44 CML patients [409]. However, no associations are between the p53 R72P genotypes, clinical parameters, and survival outcomes in a Taiwanese population [410]. Therefore, further large-scale studies appear necessary for a better understanding of the role of this SNP in CML risk and clinical outcomes.

### Chronic lymphocytic leukemia (CLL)

CLL is the most common hematologic neoplasm in the developed world characterized by the accumulation of clonal CD5^+^ B-cells in the secondary lymphoid organs -e.g., lymph nodes, spleen, blood and bone marrow [411, 412]. CLL is essentially a blood malignancy of the aging population with a median age of 72 years at diagnosis [413]. The disease exhibits remarkable clinical heterogeneity. Some patients survive for more than ten years and have a life expectancy roughly equal to that of an age-matched unaffected population without needing treatment. Conversely, others are experiencing extremely aggressive disease progression and poor outcomes despite effective chemoimmunotherapy [413–415]. This heterogeneity is partially explained by the different genetic aberrations found in CLL patients [416, 417], particularly p53 aberrations. Loss of p53 function in CLL can arise due to deletions in chromosome 17p, somatic *TP53* mutations, or a combination of both [418].

*TP53* gene defects and their association with poor survival in CLL were first described in the early 1990s [419]. Initially, the loss of the *TP53* locus was not believed to have an important role in karyotyping [420]. However, this was to change with further investigations [421]. It is now assumed that del(17p) may give rise to the loss of one of the alleles of the *TP53* gene. The incidence of *TP53* deletion is 10%-20% of all CLL cases [422, 423], albeit a little higher deletion frequency in earlier studies (up to 29%) [424, 425]. *TP53* deletion is among the significant factors that determine shorter OS [426] in CLL cases and also correlates with non-response to fludarabine and alkylating agents [423, 427], advance disease [424, 427] and progression of the disease [425]. In an investigation, in vitro fludarabine resistance was observed in CLL samples with *TP53* deletion, suggesting that in vitro drug sensitivity profiles can serve as a tool that refines individualized selection of drugs for treatment [428]. In more than 70% of CLL patients, del(17p) co-exists with *TP53* mutations in the second allele, explaining complete loss of p53 activity in these cases [422, 429–433]. Increasing evidence indicates that the co-existence of *TP53* mutations and del(17p13) correlates with worse OS, treatment-free survival (TFS), and resistance to treatment [422, 431]. *TP53* aberrations (*TP53* mutation and/or del(17p)) also predict earlier time from diagnosis to treatment compared to patients without *TP53* mutations [429].

Isolated mutations in the *TP53* gene (monoallelic mutation) without a concurrent 17p abnormality are present in 3%-6% of CLL patients [429, 432–435] and up to 18% amongst fludarabine-refractory CLL, [436], indicating a strong association between *TP53* mutations in the absence of del(17p) with rapid disease progression and poor survival in CLL. However, one study demonstrated that *TP53* mutations without del(17p) did not have an independent negative impact on progression-free survival (PFS) [437]. *TP53* mutation frequency in CLL was higher in Chinese patients (8.3%) than in other countries, intimating the importance of concurrent screening of *TP53* mutations along with del(17p) in Chinese populations [422]. Although diagnostic guidelines recommend examining CLL patients for del(17p) but not for *TP53* mutations [438], analysis of *TP53* mutations alone can help to identify an extra 3% to 6% of CLL patients with poor outcomes who should also be considered for other treatment strategies [432]. According to some literature, *TP53* mutations independently can predict rapid disease progression, short survival, and chemo refractoriness, not considerably different from patients with del (17p) [422, 429, 434]. An independent prognostic impact of *TP53* mutations on PFS and specifically OS was also reported [435]. The result of an investigation by Gonzalez et al. revealed no significant difference in response rates among CLL patients with isolated *TP53* mutation, isolated del(17p), and those with both *TP53* abnormalities. In addition, they observed a median PFS of less than six months, regardless of the presence of del(17p) in *TP53* mutant patients receiving first-line treatment compared to those without *TP53* mutations who had a median PFS of more than two years [432]. Therefore, testing for *TP53* mutation should be incorporated into the evaluation of patients with CLL before treatment initiation since it adds prognostic information notably in patients without del(17p) [429, 435, 439].

The frequency of *TP53* mutation in the majority of studies is approximately 7%-13% in patients at the time of diagnosis [422, 429, 432, 434, 435, 439–441]; however, some studies reported a higher mutation rate. The result of a study disclosed that CLL in Taiwan is distinct compared to the Western CLL. The *TP53* mutation rate at diagnosis was twice as high compared to the West (20.5% vs. 7 ~ 13% respectively), and the higher mutation rate might partly explain the poor outcomes of CLL patients in Taiwan. Therefore, *TP53* mutation analysis is valuable in evaluating the risk and prognosis of patients in a specific population [442]. Chiaretti et al. searched for *TP53* mutation in untreated CLL cases using two different methods: direct sequencing and the AmpliChip p53 Research Test. Intriguingly, by sequencing, they identified 10.2% *TP53* mutation. In contrast, the AmpliChip p53 Research Test detected 17 mutations in 14 patients (17.3%), signifying that microarray-based resequencing assay can identify a higher percentage of *TP53* mutations, although it failed to identify two microdeletions [443].

The *TP53* mutation rate is significantly higher in patients who had received prior chemotherapy with alkylating agents [444], up to approximately 25% [422, 432, 445]. It is still debatable whether *TP53* mutations are induced or selected by chemotherapy agents. Lazarian et al.’s results confirm that chemotherapy favors the selection and expansion of mutated clones rather than directly induced by therapeutic agents. They also demonstrated that among patients harboring *TP53* abnormalities at diagnosis, the acquisition of further *TP53* sub clonal alterations is more common [430]. Moreover, Dong et al. assessed *TP53* mutations in 10 CLL patients before and after treatment and concluded that *TP53* mutation could not be induced by chemotherapeutic agents [422]. Zenz et al. also suggested that detectable *TP53* mutation after treatment is selected rather than being caused by alkylating agents [446]. The incidence of *TP53* mutation also increases among cases with fludarabine-refractory CLL. Thornton et al. indicated only 7% *TP53* abnormalities in the previously untreated group, while 50% of the heavily pretreated/refractory CLL group had abnormalities detected, indicating that p53 is associated with more aggressive disease and resistance to chemotherapy [447]. Moreover, *TP53* mutation contributes to chemotherapy resistance such as chlorambucil (CLB); however, this is not the only mechanism of drug resistance in CLL, as patients harboring wild-type p53 also represent resistance to treatment [448]. Marinelli et al. examined the incidence of *TP53* mutation as well as its function (impaired p53 response to ionizing radiation (IR)) at different phases of CLL. They showed that chemoresistant CLL had the highest incidence of *TP53* mutations (21.3%) compared to progressive CLL (11.2%) and newly diagnosed cases (2.2%). In line with the molecular test results of *TP53* mutation, immunoblotting and flow cytometry detected the highest incidence of p53 dysfunctions in chemoresistant CLL (50%), progressive CLL (23.6%), and at diagnosis (5.9%). Three types of *TP53* dysfunctions have been identified in which type 1 dysfunction usually was associated with heterozygous missense *TP53* mutations at diagnosis and was partly resistant to radiation-induced killing. Types II and III, which are commonly seen in progressive and chemoresistant cases are associated with a higher incidence of microdeletions, nonsense mutations, bi-allelic *TP53* defects, and complete radioresistance. Therefore, due to the various unusual patterns of mutations in each phase of the disease, both analyses could serve as a guide to treatment choices [449].

One of the consequences of loss of *TP53* function is genetic instability [450], which is consistently correlated with complex aberrant karyotypes [429]. In a cohort of 31 relapsed/refractory CLL patients, a CK was found in 13 out of 16 patients with *TP53* mutations, while it was present in 4 out of 15 patients without *TP53* mutations, highlighting the relation between *TP53* mutation and genetic instability [430]. In addition, the result of another cohort conducted on 101 newly diagnosed CLL patients revealed a correlation between *TP53* disruption and a CK which predicted a worse OS. This suggests that karyotype analysis contributes to the risk stratification in high-risk CLL patients with highly unfavorable outcomes [451].

The p53 RNA and protein levels were also examined in CLL patients. Increased p53 expression accompanies del(17p) [452, 453], the progress of CLL [452, 454], and worse OS [452, 454]. Lin et al. reported that despite high expression levels of the p53 protein in CLL cases with *TP53* mutation, the p53 gene was under-expressed, indicating that the p53 protein commonly is regulated by post-translational rather than by transcriptional mechanisms [455]. They also showed high p53 mRNA expression in patients without *TP53* mutation [455, 456].

CLL/small lymphocytic lymphoma (CLL/SLL), accounts for almost 12% of all B-cell neoplasms. It is characterized by a monoclonal expansion of small B lymphocytes ≥ 5 × 10^9^/L in the peripheral blood and the formation of proliferation centers (PCs) in the bone marrow and lymphoid tissues. *TP53* alterations are more common in CLL patients with PCs in their BM compared to those without PCs (control group) [457]. The result of the Lenz et al. study revealed *TP53* mutation and expression in 16% and 19% of cases with prolymphocyte leukemia (PLL), respectively. They also showed that while p53 abnormalities had only affected one allele in typical CLL, 4 out of 7 CLL/PLL cases had both alleles inactivated, implying a correlation between the accumulation of p53 abnormalities and CLL/PLL developments [458].

The coexistence of *TP53* mutation with other mutations in CLL patients has also been assessed. As an example, the parallel occurrence of mutations in NOTCH1 and *TP53* genes are potent CLL progression drivers, leading to disease chemo-refractoriness [439, 459]. Kantorova et al. detected the hotspot c.7541_7542delCT NOTCH1 mutation in 17% of *TP53-mutated* CLL patients using single-cell analysis. A striking clonal heterogeneity and therapy-related clonal evolution were seen in the NOTCH1-*TP53* -mutated patients, proposing the association of concurrent occurrence of these mutations with increased risk of Richter’s syndrome [440]. *TP53* mutations together with clonal NOTCH1 conferred a shorter OS in CLL patients [441]. Another study showed an increased incidence of *TP53* mutation (16%) in the NOTCH1 mutant patients [439]. Moreover, the *TP53* mutation rate was higher in patients expressing truly unmutated immunoglobulin heavy variable (IGHV) genes than those with minimally/borderline mutated IGHV genes [460]. CLL patients with both *TP53* and IGHV mutations had significantly more favorable survival than *TP53-mutated* patients with unmutated IGHV [445].

The analysis of *TP53* codon 72 polymorphisms in CLL patients varies with latitude and race. One study suggests that the Pro/Pro genotype contributes to B-CLL leukemogenesis in the Sudanese population tenfold higher than those who have Arg/Arg genotype [461]. In contrast, in the Kochethu et al. study, the codon 72 A2/A2 genotype (homozygous arginine) was considerably common in the CLL patient and influenced the CLL susceptibility, not other biological behavior or clinical response [462]. In another investigation, codon 72 SNPs of p53 were not clinically relevant for apoptosis induction or survival in B-CLL patients [463]. Some studies report the association of the Pro72Pro genotype of rs1042522 with an increased incidence of *TP53* mutation, suggesting that *TP53* codon 72 polymorphism may serve as a risk factor for the development of *TP53* mutations in CLL [464–466]. One study specified that although the Pro/Pro genotype was correlated with an increased incidence of *TP53* mutations and deletion, it failed to impact biological tumor behavior or clinical response [464]. Other SNPs, including the C/C genotype of rs1642785 and the G/G genotype of rs2909430 (in men only), also had borderline associations with CLL risk [465]. The intron 6 polymorphism had no impact on altering susceptibility to CLL [462].

### Multiple myeloma (MM)

MM is a malignancy of terminally differentiated plasma cells and includes approximately 10% of all hematologic malignancies [467]. The disease is characterized by infiltration and accumulation of immunoglobulin-secreting neoplastic plasma cells in the bone marrow and, as a result, increased serum or urine monoclonal paraprotein levels. Clinical manifestations include lytic bone lesions, cytopenia, hypercalcemia, immunodeficiency, and renal damage [468, 469]. MM resides mainly within the bone marrow, but about 1%-2% of patients have an extramedullary disease (EMD) at the time of diagnosis, and 8% develop EMD later on in the course of the disease [469]. MM usually evolves from a premalignant, asymptomatic disorder termed monoclonal gammopathy of undetermined significance (MGUS) [470, 471], which is present in over 3% of people aged 50 years or older [472, 473]. Patients with MGUS have a lifelong risk of MM and progress to it at a rate of 1% per year [474, 475]. MM is a heterogeneous disease that is affected by a broad spectrum of genetic abnormalities [468]. *TP53* aberrations are among the reported genetic events and important markers of poor prognosis in MM [476].

Mutation of *TP53* is a rare occurrence in MM (approximately 3%-8%); however, the incidence increases in the advanced stages of the disease, implying its crucial role in disease progression [477–480]. Despite a low frequency of *TP53* mutation, their presence confers significantly worse OS compared to patients without mutation and is also associated with soft tissue plasmacytoma [477]. Moreover, *TP53* mutation is a marker of progression, even in the absence of *TP53* deletion [478]. In contrast, some studies stated a limited value of *TP53* mutation as a prognostic indicator in newly diagnosed MM (NDMM) patients and suggested that it should be confined to patients with end-stage [481]. No *TP53* mutation was observed in 51 patients enrolled in the Ortega et al. study, concluding that *TP53* mutation might be a prognostic indicator of limited value in MM [480]. Intriguingly, *TP53* is the most mutated gene (67%) in human myeloma cell lines [482]. Although Ollikainen et al. could not detect *TP53* mutations in bone marrow samples of patients with MM, they were detected in the cell lines originating from the same samples, intimating that *TP53* mutations might appear throughout the development or culture of these cell lines [483]. The presence of *TP53* mutations was significantly associated with 17p13 deletions. In Boyd et al. study, *TP53* was mutated in less than 1% of patients without del(17p) and in 27% of those carrying del(17p) [479]. These findings are consistent with another investigation in which no *TP53* mutation was reported in 38 patients without del(17p), despite 37% *TP53* mutation frequency in patients with del(17p) [484]. Almost one-third of del(17p) MM cases harbor a *TP53* mutation, which conferred an inferior prognosis for those patients with both aberrations [485]. Thanendrarajan et al. demonstrated that as the proportion of del(17p)-positive cells increases, the risk of *TP53* mutation is also raised. The adverse prognosis in this subgroup of patients is linked to biallelic *TP53* inactivation [486]. In the Chng et al. study, *TP53* mutation and 17p deletion afflicted 13% of newly diagnosed patients and emerged as an independent poor prognostic factor that could identify high-risk patients [477].

Nevertheless, deletions in 17p13 (mostly hemizygous) are a recurrent cytogenetic abnormality in MM. It constitutes an incidence rate of around 10% in newly diagnosed patients [479, 485, 487, 488] and increases up to approximately 22% across the relapsed/refractory cases [485, 489]. In an investigation conducted on a selected series of MM patients with advanced-stage disease *TP53* deletion was found in only 9% of cases [490]. However, no deletions of *TP53* were recognized in 16 MM patients either at presentation or during follow-up, proposing that *TP53* deletions are not a universal event in the development of MM but rather limited to a subclass of patients with poor prognosis [491]. *TP53* deletion is a prognostic indicator of short survival [480, 487, 488, 492, 493] and short PFS [492, 493] in patients with MM and is also associated with stage III MM disease [494, 495]. Shah et al. revealed that clonal and sub-clonal *TP53* deletions were independently associated with advanced disease and shorter OS, respectively, in MM [496]. In another study, the allelic loss of *TP53* in 12% of MM patients was considered an important factor, associated with resistance to chemotherapy [495]. Boyd et al. analyzed the outcome of 85 MM patients with del(17p) who randomly underwent either conventional or thalidomide-based induction chemotherapy. Patients with del(17p) did not have poorer response rates compared to patients without del(17p); however, del(17p) was conjoined with impaired OS. In the del(17p) group, thalidomide induction therapy was correlated with improved response rates compared to conventional therapy, but there was no influence on OS, suggesting that surrogate therapeutic strategies are a requisite for this group [479]. *TP53* deletion is also associated with poor outcomes in MM patients who underwent proteasome inhibitor-based induction followed by auto-hematopoietic cell transplant (auto-HCT). *TP53* deletion at the time of auto-HCT is related to a higher risk of progression [497]. Likewise, *TP53* deletion is an independent risk factor for shortened PFS or OS in patients with MM who receive high-dose chemotherapy and ASCT [498]. However, *TP53* deletion did not confer deleterious features in MM patients treated with total therapy 3 (TT3) as it did with TT2 [499]. Recently, cancer clonal fraction (CCF) was established as a valuable parameter in the prognostic evaluation of del(17p) in NDMM patients. Thakurta et al. suggested the assessment of a 0.55 CCF threshold and the presence of *TP53* deletion for identifying del(17p)-carrying NDMM patients with poor prognosis [500].

Plasma cell leukemia (PCL) is an aggressive form of plasma cell dyscrasia divided into primary PCL and secondary PCL and usually arises at an advanced stage of MM. Recurrent genetic changes are more frequently seen in PCL versus in MM. The incidence rate of del(17p) in PCL ranges from 35 to 50% [494, 501]. Hemizygous *TP53* deletions are also identified in 20% of primary PCL and 55% of secondary PCL [502]. *TP53* deletion is associated with a higher proportion of patients with stage IIIb/PCL [495]. The frequency of *TP53* mutations also reaches 24%-33% in PCL [501, 503]. Lionetti et al. found *TP53* mutation rates of 3.1%, 25%, and 20% in MM, primary PCL, and secondary PCL patients, respectively [478].

EMD is an uncommon manifestation in MM, characterized by the involvement of several organs, including the skin, gastrointestinal/liver, lymphatic system, respiratory tract, and central nervous system (CNS) [504]. p53 is also associated with MM cell migration, dissemination, and development of EMD [502, 505]. Chang et al. reported hemizygous *TP53* deletion in 8 out of 9 MM cases with CNS involvement, suggesting that an exceptionally high incidence of *TP53* deletions in these patients might account for metastatic features of myeloma cells [506]. Furthermore, an analysis of 834 consecutive MM patients revealed that patients with EMD at the time of diagnosis had a higher prevalence of *TP53* deletion. EMD relapse/progression is associated with EMD presentation at diagnosis as well as *TP53* deletion [507]. In a case report, a 48-year-old woman with MM had CR in BM, while extramedullary plasmacytomas were refractory to the treatment. Genetic analysis identified *TP53* deletion only in plasma cells from extramedullary plasmacytomas, suggesting that *TP53* deletion might be linked to the extramedullary plasmacytomas' lack of treatment response [508].

The incidence and prognostic significance of p53 expression have also been assessed in MM patients. Chang et al. found hemizygous *TP53* deletions in 12% of NDMM cases, strongly correlated with nuclear p53 protein expression observed in 11% of cases. Patients with p53 protein expression had significantly shorter OS than those without p53 expression [509]. p53 is also overexpressed (8.5%) in patients with NDMM [510]. In addition, aberrant p53 nuclear expression (13%) detected by IHC analysis, as well as hemizygous del(17p) (15%), were found in lenalidomide-treated relapsed/refractory MM. Patients with increased p53 nuclear expression had significantly shorter PFS and OS compared to cases without this abnormality [489]. These findings suggest that p53 IHC analysis can readily serve as a simple, rapid, and surrogate method for del(17p) in prognostic evaluation. In another investigation, the progression of MM from intramedullary to extramedullary sites was correlated with p53 nuclear expression. p53 nuclear accumulations were detected in 75% of myeloma cells derived from the extramedullary sites while only 8% of bone marrow myeloma specimens expressed p53 [511].

The association between *TP53* polymorphism at codon 72 and MM risk has been barely studied. Ortega et al. denied an influence of codon 72 allele on the pathogenesis of MM; however, the elevated Pro allele burden relates to MM progression in Brazil [512]. In another investigation, the clinical significance of *TP53* codon 72 polymorphisms was examined in Japanese patients with refractory or relapsed MM who enrolled in a prospective study of thalidomide monotherapy. *TP53* codon72 polymorphism was not associated with MM risk; however, the Pro allele was correlated with earlier relapse and shorter OS in thalidomide therapy [513]. Zmorzynski et al. suggested that P72R polymorphism might have a role in MM pathogenesis and might affect OS but not apoptosis and necrosis in cell cultures derived from MM patients [514]. Intriguingly, in a study conducted on MM patients treated with high-dose melphalan and ASCT support, OS and overall post-relapse survival were higher in cases with *TP53* SNPs compared to wild-type careers, suggesting a favorable prognostic impact of *TP53* codon72 SNP in this population [515].

### Myelodysplastic syndromes (MDS)

MDS is a very heterogeneous group of myeloid clonal disorders characterized by ineffective hematopoiesis, peripheral blood cytopenia, and clonal instability with a tendency to progress into AML [516]. The latest WHO classification of Myelodysplastic Syndromes (MDS) [274] offers a comprehensive framework for categorizing these disorders based on specific genetic abnormalities, blast count, and morphological characteristics, which are critical for prognosis and treatment strategies. MDS is first categorized by defining genetic abnormalities, such as MDS with low blasts and isolated 5q deletion (MDS-5q), which is characterized by a deletion on the long arm of chromosome 5 (5q) in the absence of other significant cytogenetic abnormalities except for one additional anomaly other than monosomy 7 or 7q deletion. This subtype shows fewer than 5% blasts in the bone marrow (BM) and less than 2% in the peripheral blood (PB) and is often associated with a favorable prognosis, especially in patients responsive to lenalidomide therapy. Another genetically defined subtype, MDS with low blasts and SF3B1 mutation (MDS-SF3B1), is characterized by the presence of an SF3B1 mutation without a 5q deletion, monosomy 7, or a complex karyotype. This subtype typically presents with ring sideroblasts, indicating a favorable outcome due to a lower risk of progression to acute myeloid leukemia (AML). In contrast, MDS with biallelic *TP53* inactivation (MDS-bi*TP53*) is characterized by less than 20% blasts in both the BM and PB, usually accompanied by a complex karyotype. This subtype involves two or more *TP53* mutations or one *TP53* mutation with evidence of copy number loss or copy-neutral loss of heterozygosity (cnLOH) at the *TP53* locus, correlating with a poor prognosis due to a high likelihood of transformation to AML [274].

In addition to these genetic abnormalities, MDS is also classified based on morphological criteria. MDS with low blasts (MDS-LB) includes cases with less than 5% blasts in the BM and less than 2% in the PB, often associated with mild to moderate cytopenias, representing a relatively lower-risk form of MDS. Another variant, hypoplastic MDS (MDS-h), is characterized by a hypocellular bone marrow and can present across various MDS subtypes, such as low-blast or increased-blast MDS, but is distinguished by its reduced cellularity. MDS with increased blasts (MDS-IB) is further divided into MDS-IB1, which includes cases with 5–9% blasts in the BM or 2–4% in the PB, and MDS-IB2, which involves cases with 10–19% blasts in the BM or 5–19% in the PB, or the presence of Auer rods, signaling a higher risk of progression to AML. MDS with fibrosis (MDS-f) is characterized by 5–19% blasts in the BM or 2–19% in the PB, accompanied by increased reticulin or collagen fibrosis in the bone marrow. This subtype often correlates with a poor prognosis due to its association with advanced disease stages and a higher likelihood of transformation to AML. Overall, the updated classification system underscores the heterogeneous nature of MDS, with genetic and morphological criteria playing crucial roles in predicting prognosis and guiding therapeutic decisions [274].

Due to various outcomes in different MDS subtypes, the International MDS Risk Analysis Workshop (IMRWS) developed a scoring system entitled the International Prognostic Scoring System (IPSS). Convenient but critical prognostic parameters in this scoring system are based on the assessment of peripheral blood cytopenia, blast percentage in the bone marrow, and cytogenetic characteristics [517]. The cytogenetics is of importance as some investigators reported a high rate of chromosomal abnormalities in primary MDS. Patients with MDS are divided into three categories: CK (> 3 abnormalities), NK, and balanced chromosomal abnormality. The most frequent chromosomal abnormalities are monosomy 7 (-7) or 7q-, 5q-, and trisomy 8 (+ 8) [518]. Treatment is selected according to the risk stratification, transfusion needs, bone marrow blasts percentage, cytogenetic, and mutational status of patients and includes growth factor support, lenalidomide, hypomethylating agents, chemotherapy, and allo-SCT [519].

*TP53* has been identified as one of the driver mutations in MDS pathogenesis. *TP53* mutations occur in approximately 8%-14% of de novo MDS patients [307, 520–522], whereas it increases in therapy-related MDS (t-MDS), approaching 38% [304, 306, 521–523]. The presence of *TP53* mutations has long been identified as a predictor of poor prognosis, lower OS [522, 524–526], lower PFS [526], and progression to AML [522, 525, 527]. In a large cohort of patients with hematological malignancies, the presence of a *TP53* mutation alone can reduce OS drastically in MDS patients [319]. It was also suggested that *TP53* mutation is a terminal genetic event in the development of MDS since mutations were detected in four out of eight cases of advanced MDS subtypes (refractory anemia with excessive blast, and AML from MDS), but not in 16 cases of the less advanced subtypes [528]. Kita-Sasai et al. revealed that patients with the *TP53* mutation have a significantly poor prognosis regardless of the IPSS subgroup they belong to [529]. In a cohort of 38 t-MDS/AML patients, *TP53* mutation and loss of the *TP53* locus occurred in 21% and 15% of patients, respectively, which were correlated with worse OS compared to those with wild-type *TP53* [304]. A higher *TP53* mutation rate was also affirmed in t-MN compared with de novo MDS (37% vs. 14.5% respectively), accompanied by inferior outcome. However, the mutation type, pattern, distribution of mutated loci, and median mutational allelic frequency in *TP53* were not different between do novo and therapy-related disease, implying the occurrence of *TP53* mutation at an early stage t-MN disease and participation of other factors in the further development of t-MN [306]. Pediatric MDS (PMDS) is rare and accounts for less than 10% of childhood hematologic diseases [530]. In a group of PMDS, Jekic et al. reported the absence of *TP53* mutations in 35 patients [531]. Although they suggested that *TP53* mutations were not critical in the evolution of PMDS, Silveira et al. indicated a strong involvement of *TP53* deletions in PMDS pathogenesis and their value as candidates for molecular markers in PMDS [532]. Moreover, among nine children with MDS, one patient exhibited deletion of the *TP53* gene in one allele and mutation in the other allele, suggesting that biallelic inactivation of the *TP53* gene might contribute to the occurrence of MDS [533].

A statistically significant relationship is between *TP53* mutation and CK [524], in which more than 75% of MDS cases with *TP53* mutations have CK [534–537]. In a large cohort of CK-MDS patients, *TP53* mutation status was the most significant risk marker. This indicates that cytogenetics alone is not sufficient for the prognostic evaluation of CK-MDS patients, and assessment of *TP53* mutations should also be considered in this population [538]. MDS patients with CK have a poor prognosis regardless of the treatment [536]. The outcome of post-HCT is more favorable in *TP53-mutated* patients without CK than in *TP53-mutated* patients with CK [535, 539]. In contrast, the incidence of del(17p) is not very common (up to approximately 5%) in MDS [540], and it is asserted that abnormalities of 17p do not take a great part in both the pathogenesis of MDS and its progression to AML [541]. Patients with i(17q) also present with infrequent *TP53* mutations [524].

*TP53* mutations are not the only prognostic feature in MDS patients, and several researchers have investigated the VAF of *TP53* mutations influence on survival rate. In low-grade MDS and MDS with a non-CK, *TP53* mutations have a lower VAF and as a result, a better survival [542]. In the Belickova et al. study, *TP53* mutations were detected in 13% of lower-risk MDS patients. They also revealed that patients with a mutational burden of < 6% VAF were stable for long periods without progression and adverse prognostic impact on PFS or OS. Therefore, it can be speculated that the clinical influence of *TP53* mutations in lower-risk MDS patients depends on the level of mutational burden [526]. *TP53* VAF could also stratify distinct prognostic groups irrespective of clinical prognostic scoring systems [543]. Together, these observations suggest that VAF should be incorporated into the management of patients with MDS.

Hypomethylating therapies (HMT), including azacitidine (AZA) and decitabine (DAC), are the first-line therapy in high-risk MDS [519]. Although *TP53-mutated* MDS patients primarily respond robustly to HMA, the duration of response is considerably shorter than wild-type patients [544]. *TP53* mutations are also the only predictors of DAC-induced complete responses. Likewise,10 of 15 patients (66.7%) who harbored *TP53* mutations achieved a CR; though not associated with longer survival due to early relapses and AML transformation [545]. The analysis of *TP53* mutations in 62 patients with high-risk MDS treated with AZA revealed its prognostic role for poor survival but not for the response to treatment [537]. Among 107 Korean patients with MDS who underwent HMT, *TP53* mutation was an independent predicting factor of poor prognosis [546]. Allo-SCT is another potential curative treatment for high-risk MDS patients; albeit with high mortality after transplantation [547]. The influence of *TP53* mutation on transplantation outcomes were investigated in several studies. Kim et al. reported that in de novo MDS patients who received HCT, *TP53* mutation was correlated with a higher CIR but not non-relapse mortality [520]. The frequency of *TP53* mutation in patients with MDS receiving HMT or SCT was 7.7%, associated with a significantly poor survival rate [548]. *TP53* mutations are also identified as the most powerful predictor of poor OS after transplantation amongst patients with MDS [521, 549]. Patients with both *TP53* mutations and CK have particularly poor post-HCT outcomes with frequent early relapse; therefore, alternative therapies should be taken into consideration for these patients [539]. *TP53*-mutated patients with CK represent a distinct subset of myeloid malignancies who are unlikely to benefit from transplantation, whereas cases with *TP53* mutations alone (without CK) have a significantly more favorable survival post-transplant [535]. Ciurea et al. showed that not all *TP53* -mutated patients with MDS who received their first ASCT had poor outcomes. They illustrated that *TP53* -mutated patients with low HCT-comorbidity index (HCT-CI), good performance status, and disease in first or second CR (CR1/2) at the time of transplantation did significantly better with this procedure and achieved a long-term survival [534]. Aldoss et al. demonstrated that *TP53* mutation was not detrimental in patients with t-MDS who underwent allo-HSCT and they had similar outcomes compared to the transplanted patients with de novo MDS with *TP53* mutations. Despite a 30% incidence rate for *TP53* mutation in t-MDS, it did not adversely affect transplant outcome and patients could be cured with allo-HSCT [523].

The del(5q), either isolated or with additional chromosomal abnormalities, is the most frequently reported abnormality in MDS and characterizes a subclass of patients with lower-risk MDS [550]. Additional aberrations such as *TP53* mutation indicates poor prognosis and, especially, progression into AML. In Fidler et al. study, five different *TP53* mutations were seen in 3 out of 40 (7.5%) patients with MDS who had a del(5q). All three patients with *TP53* mutation were diagnosed with refractory anemia with excessive blast, suggesting that *TP53* mutation is related to the more advanced MDS subtypes [551]. Lodé et al. screened for *TP53* mutations in a cohort of MDS patients receiving lenalidomide in the course of their treatment. They detected *TP53* mutation in 25% of patients at diagnosis. In half of the patients negative at diagnosis, *TP53* mutations arose during the course of the disease and in 37.5% of cases, *TP53* mutations were not found at any time. Therefore, in lower-risk del(5q) MDS, disease progression is commonly associated with the evolution of pre-existing or emerging subclones harboring a *TP53* mutation [552]. A negative impact for *TP53* mutations on the survival of patients with MDS del(5q) as well as the lower sensitivity of *TP53* mutated clones to lenalidomide treatment was also reported in a recent cohort [553]. Jadersten et al. observed *TP53* mutations in 10/55 (18%) patients with low and intermediate-1 risk MDS carrying del(5q), which render them at higher risk for disease evolution to AML [527] Another investigation reported *TP53* mutations in 17% of lower-risk MDS with isolated del(5q),associated with shorter survival [554]. Moreover, loss of the long arm of chromosome 5 due to unbalanced translocations associates with *TP53* mutation, clonal evolution, and poor prognosis in MDS [555]. Christiansen et al. displayed the company of *TP53* mutations with deletion or loss of 5q and poor prognosis in t-MDS /t-AML cases after previous treatment with alkylating agents [556]. Intriguingly, in a recent retrospective study of on 63 MDS patients having del(5q), *TP53* mutations were present in 23.8% of cases. Despite the generally poor prognosis associated with *TP53* mutations in MDS, no significant difference in overall survival was observed between *TP53*-mutant and wild-type patients in this cohort. A majority of patients with del(5q) MDS received lenalidomide, with notable responses including hematologic improvement in 37.2% and no response or disease progression in 62.8%. The median overall survival for patients treated with lenalidomide varied significantly based on response, highlighting its impact on outcomes [557]. These findings suggest that while *TP53* mutations are typically linked to worse outcomes in MDS, the del(5q) subset may behave differently, underscoring the need for further research to understand the implications of *TP53* mutations and treatment responses in this specific group.

Additionally, a 2024 study reported on a patient with MDS-del(5q) who developed *TP53* mutations during lenalidomide therapy. This case confirmed previous findings that *TP53* mutations can emerge or expand during treatment [552]. The patient, initially responsive to lenalidomide, experienced a relapse with new *TP53* and RUNX1 mutations, leading to the discontinuation of lenalidomide. Salvage therapy with a 3-day course of decitabine, as opposed to the standard 5-day regimen, successfully restored transfusion independence, achieved cytogenetic remission, and eliminated the *TP53* mutation. This case highlights lenalidomide’s potential to select for *TP53* mutant clones and emphasizes the importance of ongoing molecular monitoring and timely transition to hypomethylating agents like decitabine. The authors recommend performing next-generation sequencing (NGS) at diagnosis and periodically during lenalidomide therapy to detect emergent *TP53* mutations. For cases with treatment-emergent *TP53* mutations or clonal expansion, transitioning to hypomethylating agents and monitoring *TP53* variant allele frequency (VAF) is advised. These strategies are crucial for bridging therapy before allogeneic stem cell transplantation, the only long-term treatment option for MDS-del(5q) and *TP53*-mutated myeloid neoplasms. Further research is needed to explore the potential benefits of combining hypomethylating agents with venetoclax [558].

p53 is overexpressed in BM biopsies of almost 10%-25% of primary MDS [559, 560] and in approximately 30% of t-MDS [336]. Overexpression of p53 protein detected by IHC is coupled with *TP53* mutation [316, 336, 559], worse OS [316, 336, 560], risk of leukemia progression [559, 561] and chromosomal abnormalities [316, 336, 559]. High p53 protein expression was observed in 35% of patients with lower-risk del(5q) MDS treated with lenalidomide which was correlated with AML transformation, lower cytogenetic response to treatment, worse OS and presence of *TP53* mutation [562]. Ruzinova et al. stated that p53 IHC is a reliable proxy for both the *TP53* mutations status as well as mutation clearance in DAC-treated MDS [563]. p53 expression can also serve as a prognostic factor in MDS patients treated with AZA, since OS was significantly lower in p53 -positive patients compared to p53—negative patients [564]. Nevertheless, Müller-Thomas et al. revealed that p53 expression did not negatively affect treatment response [565]. Analyzing patients with de novo MDS exhibiting fibrosis (MDS-F) revealed high p53 expression, accompanied by higher BM blast counts, alterations of chromosomes 5 or 7, CK, high- and very-high risk IPSS-R groups, *TP53* mutations, and shorter OS [566, 567]. p53 immunostaining can also be used in the differential diagnosis, including differentiating hypocellular MDS (hMDS) from other causes of bone marrow failure such as aplastic anemia (AA) [568]. Cha et al. reported higher p53 -positive BM cells among patients with hMDS than cases with AA [569]. The result of the Elghetany et al. study revealed p53 protein overexpression in hyperplastic refractory anemia (79%) and hypoplastic refractory anemia (57%). In contrast, no nuclear staining is detected in biopsies of AA patients and of normal individuals. Hence, they considered that p53 mechanism in the pathogenesis of MDS is totally different from AA and p53 IHC staining of BM biopsy provides valuable information for discriminating between these diseases [570]. Overall, the identification of p53 protein expression levels has a growing clinical utility.

### Lymphoma

Lymphoma represents a large group of malignant neoplasms arising from immune system components such as T, B, and natural killer (NK) cells. It consists of two major subgroups: Hodgkin lymphoma (HL) and non-Hodgkin lymphoma (NHL). HL includes about 10% of all lymphomas and tends to spread contiguously from one group of lymph nodes to the sites along the lymphoid system [571]. However, NHL can impact different parts of the body, including the liver, bone marrow, and spleen. Lymphomas are classified according to the WHO category, which discriminates lymphoid neoplasms derived from precursor lymphoid cells from those developed from mature lymphoid cells and further divides each group into neoplasms of B-cell or T-cell origin [572].

NHLs are a heterogeneous group of B, T, and NK cells neoplasms that arise from both mature and precursor cells in the lymph nodes but can also extend to other organs [573]. Several studies examined p53 alterations in different subgroups of NHL. The majority of *TP53* mutations have been reported in exons 5–9 in different histological subsets of NHLs. *TP53* mutations occur mostly in intermediate [574] and high-grade [575] B-cell NHLs. Therefore, it is hypothesized that *TP53* mutations might have an association with tumor progression. In Chen et al. study, 50% of patients who carried *TP53* point mutations achieved CR after chemotherapy but relapsed after 6–23 months and the remaining 50% died due to refractoriness to chemotherapy [574]. Moreover, *TP53* mutations are associated with poor prognosis irrespective of treatment in elderly patients with aggressive B-NHL [435]. A meta-analysis result confirmed the relation between *TP53* mutations and poor prognosis in NHLs [576]. Intriguingly, most of the cases with *TP53* missense mutations, but not nonsense mutations, have p53 protein overexpression [577, 578]. p53 expression is a common finding in high-grade lymphoma [575] and associates with short survival and resistance to chemotherapy [579]. In one study, 9 of 13 cases (69.2%) with NHLs of the head and neck expressed p53 protein and 7 (53.8%) harbored gene mutations. This indicates a relationship between the high frequency of p53 mutations and histological malignancy [580]. The expression of p53 can also predict treatment failure in aggressive NHLs. Navaratnam et al. reported that 50% of patients who had p53 positive tumors achieved CR as opposed to 77% of patients with p53 negative tumors. In addition, 26% of the p53 negative group relapsed, and this rate increased to 60% in the p53 positive group [581]. Analysis of p53 Arg72Pro polymorphism revealed that subjects with p53 Pro72 demonstrated a significantly higher risk of NHL in Japanese [582], Chinese [583] and Korean [584] populations.

Diffuse large B‐cell lymphoma (DLBCL) represents the most common type of aggressive NHL, and is a heterogeneous group of diseases both clinically and biologically. Frequently recurring mutations are characteristics of DLBCL, and several studies examined *TP53* mutations in this group of patients [585]. In general, the frequency of *TP53* mutation in DLBCL is 10%-23% [577, 586–589]. Mutations in the *TP53* DBDs are also the strongest predictors of poor OS [590]. In 102 de novo DLBCL, 13 patients (12.7%) displayed *TP53* mutations, which correlated with inferior survival [586]. Similarly, Kerbauy et al. identified 8 *TP53* missense mutations in 6 out of 48 DLBCL cases (12.5%), associated with an adverse OS, but not with a remission rate after initial chemotherapy. Moreover, they showed that of the six patients with a *TP53* missense mutation, 5 cases had p53 overexpression [587]. Hiyama et al. stated that mutations of the *TP53* gene might be the causative agent of gastric DLBCL [591]. *TP53* mutations were also detected in 23% of low and low-intermediate-risk DLBCL samples, associated with poor prognosis. Hence, this implies the importance of the routine p53 evaluation in those who need alternative treatment [592]. *TP53* mutations are more frequent in DLBCL with MYC translocations compared to DLBCL without MYC translocation (42.86% vs. 11.5%, respectively). Additionally, loss of *TP53* is observed in most cases, which is an independent unfavorable predictor of clinical outcome [589, 593]. p53 alteration was also associated with relapse of de novo DLBCL, as 2 out of 5 (40%) cases with normal p53 at diagnosis presented p53 alterations at relapse [594]. The result of whole-exome sequencing for six patients with refractory and seven with responsive DLBCL revealed 50% *TP53* mutation exclusively in refractory cases accompanied by copy number deletions [588]. Simonitsch-Klupp et al. assessed the *TP53* deletion in a subgroup of DLBCL, called lymphomas with plasmablastic/plasmacytoid features (PB/PC-Fs). By FISH analysis, they found 85% monoallelic *TP53* deletion in PB/PC-F, representing resistance to standard chemotherapy and shorter OS [595]. Overexpression of p53 is also found in 60% of DLBCL cases, and the majority of them have wild-type p53. There is no difference in OS or DFS between both groups of patients with normal and increased p53 expression [587]. In contrast, in another study, p53 protein overexpression is detected in more than 80% of tumor cells harboring a *TP53* missense mutation [577]. Patrascu et al. suggested a correlation among p53 positively, low prognostic index, and poor survival rate, making them helpful diagnostic and therapeutic targets for DLBCL [596]. Survival analysis also demonstrated that p53 overexpression predicted worse PFS and OS [589]. The *TP53* codon 72 polymorphisms have also been assessed in DLBCL. SNP72 G/C did not influence DLBCL onset or survival in central European Caucasians [597]. Likewise, Zainuddin et al. reported that codon 72 genotypes had no impact on the prognosis and survival of DLBCL patients and hence, it seemed to lack clinical relevance in this type of lymphoma [586]. Nonetheless, *TP53* Arg72 is a favorable prognostic factor in Chinese DLBCL patients who received cyclophosphamide, doxorubicin, vincristine, and prednisone (CHOP) or CHOP-like as frontline regimen [598].

Mantle cell lymphoma (MCL) is an aggressive and rare type of NHL. Several molecular markers have been recognized as feasible prognostic factors in MCL, including *TP53* mutations. The *TP53* mutation is an independent negative prognostic marker for MCL. Stefancikova et al. found high *TP53* mutation frequency (9 of 33 patients) in MCL tumors. The difference in OS between MCL patients carrying the *TP53* mutation and patients without the mutation was considerable (17.6 months vs. 64.8 months respectively) [599]. Within 32 cases with a leukemic presentation of MCL, 6 (18.8%) had *TP53* mutation. However, the mutation was not sufficiently an independent prognostic factor in patients with MCL at an advanced stage [600]. Both *TP53* mutation and CK could independently portend a dismal prognosis in MCL cases. The combination of these two markers could help to stratify patients more precisely into 3 prognostic groups with the worst outcome in individuals with CK and *TP53* mutation [601]. Furthermore, *TP53* mutations could identify a phenotypically distinct and highly aggressive MCL entity with inferior or no response to treatments such as cytarabine, rituximab, and ASCT. Hence, it is suggested to classify MCL patients according to *TP53* mutational status and they should undergo separate clinical trials [602]. The loss of the p53-specific locus 17p has also been analyzed in MCL. Less than approximately 50% of MCL cases with *TP53* mutation have a concomitant 17p deletion [599, 603]. In a small cohort of MCL patients, only 9.1% of all cases had *TP53* deletion [599], whereas the frequency of deletion of the *TP53* locus reached 32% in a larger cohort of MCL [603]. However, in both cohorts, the absence of 17p deletions did not influence survival. There are two major variants of MCL: nodal MCL (N-MCL), which affects the lymph nodes, and leukemic variant (L-MCL), which encompasses the bone marrow, peripheral blood, and the spleen. Leukemic non-nodal MCL is usually an indolent disease; however, a relatively aggressive clinical course was identified in 3 cases with *TP53*, ATM, and/or 13q14 deletions [604]. Sakhdari et al. examined *TP53* mutation status in 21 patients with N-MCL and 5 with L-MCL using NGS. 6 of 21 (28.5%) N-MCL cases harbored *TP53* mutations which all of them were clonal. Of 5 L-MCL cases, 3 (60%) carried *TP53* mutation, with these mutations being sub clonal in 2 out of 3 mutated patients. Therefore, they suggested monitoring these patients for expansion of *TP53* mutated clones, as identification of clonal expansion in L-MCL patients indicates the need for therapeutic intervention despite low tumor burden [605].

Follicular Lymphoma (FL) is the second most common NHL type and typically is an indolent disease. O’Shea et al. detected heterozygous *TP53* mutation in 6% of FL cases, associated with older age, higher International Prognostic Index score, shorter PFS, and OS [606]. Some studies assessed the role of *TP53* mutation in the transformation of FL. Møller et al. found that relapse of FL was related to p53 alterations since 5/6 (83%) FL cases with normal p53 at diagnosis displayed p53 alterations at relapse [594]. Similarly, *TP53* mutation contributes to the blastic transformation in FL patients with double rearrangement at the bcl-2 locus (mbr/vcr) [607]. These results recommend the incorporation of *TP53* screening into the design of clinical trials in FL.

Hairy cell leukemia (HCL), hairy cell leukemia variant (HCL-V), and splenic marginal zone lymphoma (SMZL) are splenomegalic B-cell disorders with overlapping features, but each follows a distinct clinical course and respond differently to treatment [608, 609]. Surprisingly, a high rate of *TP53* mutations (28%) was detected in HCL, as in other NHLs, which predicted poor treatment outcomes [610]. A high incidence of *TP53* deletion was reported in both HCL and HCL-V (75% and 100%, respectively). However, the proportion of cells with *TP53* deletion in HCL-V cases was significantly higher than that in HCL cases, and more than half of the HCL-V patients with greater than 22% of cells with *TP53* deletion presented features of disease progression [611]. Hockley et al. identified partial or complete deletions of 17p, including *TP53* in 5/15 HCL-V cases, but not in HCL. They also found that loss of *TP53* might result in greater genomic instability as well as the chemo-refractoriness of HCL-V [612]. Mutation analysis of *TP53* also showed a significantly higher frequency of mutations in HCL-V (30%) compared to SMZL (8%). In HCL-V, survival at five years was shorter in subjects with a *TP53* mutation than those without. Therefore, the inferior outcome in HCL-V compared to SMZL, to some degree, might be attributable to the higher frequency of *TP53* mutations in HCL-V cases versus in SMZL [613]. SMZL, also described as splenic lymphoma with villous lymphocytes (SLVL), is a rare chronic subtype of B-cell disorder that involves the spleen, BM, and peripheral blood [608]. A study has shown that among 12 cases with SMZL transforming to large B-cell lymphoma, p53 inactivation was detected in only one case [614]. In an investigation of 20 SMZL subjects, Sol Mateo et al. found two cases with *TP53* mutation [615]. In a multinational cohort of 175 SMZL patients, Parry et al. discerned 16% recurrent mutations in *TP53* and declared that *TP53* gene mutation was an independent marker of reduced OS [616]. In a study of 59 SLVL cases, 11 individuals had p53 abnormalities (8 with hemizygous *TP53* deletion alone, 2 with both hemizygous *TP53* deletion and protein expression, and 1 with protein expression alone) which was associated with a more aggressive disease course [617]. Conversely, Baldini et al. observed a higher *TP53* mutation frequency (40%) in nonvillous SMZL patients who were associated with poor prognosis. However, it should be taken into account that this study from 1994 incorporated cases with CD5 and/or CD23 expression into their analysis which would not have been included in a more recent series of SMZL [618].

Burkitt lymphoma (BL) is a highly aggressive and rapidly growing B-NHL, identified as the most common histologic subtype in children. Among all types of lymphoma, BL has a higher frequency of *TP53* mutations. However, much of the published data is based on cell lines [619, 620] and there is a paucity of information in the literature regarding the role of *TP53* mutations in the pathogenesis or the development of BL. Bathia et al. detected 37% of *TP53* mutations in BL cases from South America, although there was no reference to the number of included children and whether they represented newly diagnosed or relapsed disease [621]. These results are in line with the reports of other authors, who found a 33% *TP53* mutation rate in BL [419, 622]. Concerning these findings, *TP53* mutations are quite frequent in BL and may contribute to lymphomagenesis or disease progression. Nevertheless, one study revealed a lower incidence of *TP53* mutation (19%) in newly diagnosed BL cases. Analysis of *TP53* point mutations suggested that they usually consisted of missense mutations and were not associated with poor response to intensive therapeutic regimens [623]. Likewise, Klumb et al. detected mutations of *TP53* in 20% of children with BL. They demonstrated that certain types of mutants could alter the protein distinctly and a more aggressive phenotype is speculated. No statistically significant difference was perceived in EFS between patients with and without the mutation [578].

Mucosa-associated lymphoid tissue (MALT) lymphoma is defined as a low-grade lesion with the potential to turn into a high-grade lymphoma. Mutations of *TP53* were identified in 2/11 (18.1%) individuals with high-grade MALT-lymphomas, while they were negative (0/16) in all patients with low-grade histology [624]. In Du et al. study, *TP53* mutations together with allelic loss are more prevalent (67%) in high-grade forms of extranodal than that in low-grade tumors (9%) [625]. Zhang et al. investigated *TP53* deletion in 50 primary gastrointestinal MALT lymphoma patients. They found that the p53 gene deletion rate in stage III-IV patients was significantly higher (54.5%) compared to stage I-II patients (22.6%). This indicates the p53 deletion role in the progression of primary gastrointestinal MALT lymphoma [626]. Moreover, IHC analysis of primary gastric MALT lymphomas revealed that p53 protein expression was expressed in 72% of cases, and its positivity increased as the histological grade advanced. This suggests that p53 might lead to the transformation from low-grade to high-grade gastric lymphomas [627].

*TP53* abnormalities were also investigated in mature T and NK neoplasms. By SSCP, *TP53* mutation was detected in 47.6% of patients with nasal NK/T-cell lymphoma, which gave an insight into its role in the lymphomagenesis of nasal NK/T-cell lymphoma [628]. In another investigation, *TP53* missense mutation occurred in 19% of cases with nasal NK/T-cell lymphoma with a prognostic indicator for an aggressive course of the disease [629]. *TP53* mutation was also observed in 24% (5 of 21) of cases with nasal NK/T-cell lymphoma from Mexico, associated with large cell morphology and advanced-stage disease [630]. A relatively high incidence (23%-56%) of p53 protein overexpression is also reported in nasal NK/T-cell lymphomas [628–630]. In Quintanilla-Martinez et al.’s investigation, the proportion of p53 overexpressing cells was lower compared to other studies (23%), and it was linked to *TP53* mutations [630]. The result of a study conducted on peripheral mature T and NK cell lymphomas disclosed the occurrence of *TP53* mutations, particularly in patients with 72Pro homozygous genotype, associated with poor prognostic features in this subset of lymphoma. Additionally, overexpression of p53 was found in 11 of 56 (19.6%) tumors which was a reliable indicator of *TP53* mutation [631]. The most common cutaneous T-cell lymphomas (CTCL) are mycosis fungoides (MF) and Sézary syndrome (SS). Gros et al. detected *TP53* mutations and deletion in 29% and 84% of patients with either primary or secondary SS, respectively. However, no difference in prognosis was perceived with respect to *TP53* status [632]. In addition, p53 overexpression was observed in 10 out of 15 patients with CTCL who underwent large cell transformation (LCT), but no mutations were identified in 6 patients positive for p53 expression, highlighting that overexpression of p53 protein in LCT and disease progression of CTCL might be due to other mechanisms rather than *TP53* mutation [632]. Adult T-cell Leukemia/Lymphoma (ATLL) is a human neoplasm caused by the retrovirus HTLV-1. The incidence of *TP53* mutations is higher in acute subtypes of ATLL than in chronic, indicating that *TP53* mutations may play an important role in the transition from chronic to acute ATL [633, 634]. Tawara et al.’s results demonstrated that p53 genetic alterations influenced tumor cell character which is enough to reduce the patient’s survival period [635]. Two studies demonstrated a low incidence of *TP53* mutation (4.5% and 8%) in anaplastic large-cell lymphoma (ALCL) [575, 636]. Another survey revealed that all of the ALCL tumors with *TP53* mutation overexpressed p53. Therefore, the high frequency of p53 expression in ALCL besides the low frequency of p53 gene mutations raises the possibility of the existence of another mechanism to stabilize the p53 protein [636].

HL is an uncommon B-cell lymphoid malignancy. No clearly defined risk factors have been recognized for the development of this disease; hence, identifying poor prognostic features can improve risk‐adapted therapy [637]. Spector et al. analyzed p53 expression in 83 patients with HL but did not find any association between p53 expression and CR rate, FFS or OS [638]. Moreover, in a cohort of 44 HL patients with second malignant neoplasms, *TP53* was not frequently mutated [639]. Although not much information is available, it is unlikely that p53 alterations have a role in the pathophysiology of the HL. The details are presented in Table 5.

**Table 5:** Summary of studies regarding TP53 Alterations and Their Prognostic Impact Across Hematological Malignancies

Malignancy	Subtype/Phase	Frequency of TP53 Point Mutations	Frequency of 17p Deletions	Multi-Hit TP53 Alterations	Prognostic Impact	References
**AML**	**De Novo AML**	~ 10%	< 5%	Rare	Poor outcomes, resistance to chemotherapy	[284–286]
	**Therapy-Related AML (t-AML)**	21%-38%	40%-75%	Common	High risk, poor prognosis	[304–307]
	**Secondary AML (s-AML)**	22%-27.3%	Variable (e.g., 53.6% in MRC)	Frequently present	Inferior outcomes, shorter OS	[312–315]
	**Complex Karyotype (CK) AML**	53%-78%	40%-75%	Often associated	Extremely poor prognosis, resistance to therapy	[281, 288–293]
	**Monosomal Karyotype (MK) AML**	Varies, often present	53.60%	Commonly observed	Worst prognosis among AML subtypes	[291, 318]
	**Acute Megakaryoblastic Leukemia (AMKL)**	Rare, but present	Rare	Rare	Prognosis varies; debated role of TP53 mutations	[302, 303]
	**AML with Myelodysplasia-Related Changes (MRC)**	Variable	High frequency	Common	Associated with high-risk disease, inferior OS	[311–316]
**MDS**	**MDS with 5q Deletion (MDS-5q)**	7.5% (related to advanced cases)	Rare (< 5%)	Rare	Generally favorable prognosis, especially with lenalidomide	[550–552]
	**MDS with SF3B1 Mutation (MDS-SF3B1)**	Low, specific mutation type	Rare	Rare	Favorable prognosis, low risk of progression to AML	[274, 516]
	**MDS with biallelic TP53 Inactivation (MDS-biTP53)**	20% +	Rare	Common	Poor prognosis, high likelihood of transformation to AML	[274, 516]
	**Hypoplastic MDS (MDS-h)**	Variable, generally low	Rare	Rare	Relatively lower risk, prognosis varies	[274]
	**Increased Blasts MDS (MDS-IB)**	Varies by subtype (IB1, IB2)	Rare	Rare	Higher risk of progression to AML, worse outcomes	[274]
	**MDS with Fibrosis (MDS-f)**	Elevated in advanced cases	Rare	Common	Poor prognosis, often associated with advanced disease	[274]
	**Complex Karyotype MDS (CK-MDS)**	75% +	Rare	Common	Extremely poor prognosis	[536–538]
**CML**	**Chronic Phase (CP)**	Rare (1–5%)	Rare (< 5%)	Rare	Generally favorable prognosis with TKI treatment	[384–395]
	**Accelerated Phase (AP)**	10–25%	25% +	Common	Increased risk of progression; poorer prognosis	[392, 396]
	**Blast Phase (BP)**	15–30%	30–40%	Common	Very poor prognosis, resembles acute leukemia	[387–394]
	**Myeloid Blast Crisis**	Elevated in myeloid cases	Common (associated with BP)	Common	Aggressive disease course, worse outcomes	[392, 396]
	**Lymphoid Blast Crisis**	Rare	Rare	Rare	Prognosis varies, generally better than myeloid BP	[392, 394]
	**Complex Karyotype CML**	High prevalence (up to 75%)	Common	Common	Extremely poor prognosis	[387–397]
**ALL**	**Childhood ALL**	13% (newly diagnosed), 29% (relapsed B-cell), 46% (relapsed T-cell)	9% (B-ALL), 18% (T-ALL)	Common in relapsed cases	Inferior outcomes in relapsed cases, critical for remission	[340–349]
	**Adult ALL**	8.2% (at diagnosis), 15% (newly diagnosed)	Common	Common	Correlated with worse overall survival and higher relapse	[350–354]
	**Philadelphia-negative Adult ALL**	8% (associated with worse outcomes)	Rare	Rare	Chemoimmunotherapy may mitigate poor prognosis	[350–354]
	**Low-Hypodiploid ALL**	91.2% (childhood), 90.9% (adult)	Common	Common	Extremely poor prognosis, often germline mutations	[355–361]
**CLL**	**CLL with del(17p)**	N/A	10%-29%	N/A	Shorter overall survival (OS), non-response to treatments	[422–427]
	**CLL with *TP53* mutation**	3%-6% (up to 18% in fludarabine-refractory cases)	N/A	N/A	Indicates poor survival, rapid disease progression	[429, 432–435]
	**CLL with del(17p) and TP53 mutation**	Common (70% co-existence)	Common	N/A	Worse OS, increased treatment resistance	[422, 429–433]
**Lymphoma**	**Diffuse Large B-cell Lymphoma (DLBCL)**	10%-23%	Common	Often with MYC translocations	Poor OS	[577, 586–589]
	**Mantle Cell Lymphoma (MCL)**	High frequency	32%	TP53 mutation + CK	Poor OS	[599, 603]
	**Follicular Lymphoma (FL)**	6%	Rare	Related to blastic transformation	Poor PFS/OS	[606, 607]
	**Hairy Cell Leukemia (HCL)**	28%	75%	Higher in HCL-V	Poor outcomes	[610, 611]
	**Burkitt Lymphoma (BL)**	19%-37%	Rare	Often with other mutations	Variable	[621–623]
	**Mucosa-Associated Lymphoid Tissue (MALT)**	18.10%	Higher in high-grade	Often in high-grade forms	Progressive	[624–627]
	**Nasal NK/T-cell Lymphoma**	19%-47.6%	Common	Frequently with large cell morphology	Aggressive	[628–630]
	**Mycosis Fungoides (MF)**	29%	84%	Linked to transformation	Variable	[632]
	**Adult T-cell Leukemia/Lymphoma (ATLL)**	Higher in acute	Rare	Higher mutation rate in acute	Poor survival	[633, 634]
	**Hodgkin Lymphoma (HL)**	Rare	Uncommon	Not prominently involved	No significant correlation	[637, 639]
**Myeloma**	**Multiple Myeloma (MM)**	3%-8%	10%-22%	Poor OS and PFS with TP53 aberrations	Associated with advanced disease	[467–469, 476, 477]
	**Plasma Cell Leukemia (PCL)**	24%-33%	35%-50%	Aggressive, linked with stage IIIb/PCL	Higher mutation rates than MM	[494, 501–503]
	**Extramedullary Disease (EMD)**	Rare	Higher prevalence in EMD patients	Poor prognosis related to TP53 deletion	Linked to treatment resistance	[504–507]
	**MGUS**	Rare	N/A	N/A	N/A	[470, 474]

*AML* Acute myeloid leukemia, *MDS* Myelodysplastic syndrome, *CML* Chronic myeloid leukemia, *ALL* Acute lymphoblastic leukemia, *CLL* Chronic lymphocytic leukemia, *OS* Overall Survival, *PFS* Progression-free survival, *MGUS* Monoclonal gammopathy of undetermined significance, *N/A* Not available

### Clonal Hematopoiesis

Clonal hematopoiesis is defined by the clonal expansion of hematopoietic stem and progenitor cells (HSPCs) that harbor detectable somatic mutations [640]. Sequencing studies involving large cohorts of healthy individuals have revealed that the prevalence of clonal hematopoiesis progressively increases with age, leading to the concept of age-related clonal hematopoiesis [641]. The understanding of clonal hematopoiesis (CH) and its characteristics is rapidly advancing alongside improvements in sequencing technologies. As a result, the detection of CH is becoming an increasingly important aspect of clinical practice. This growing awareness of CH intersects with established diagnostic pathways, creating 'grey zone' scenarios for clinicians. Interpreting genomic data can be a significant clinical challenge in certain cases, complicating the diagnostic process. The distinctions between various entities are often subtle [640].

In a recent study [642], researchers examined the relationship between clonal hematopoiesis (CH) and rare pathogenic or likely pathogenic (P/LP) germline variants in patients with solid tumors. Utilizing prospective tumor-blood paired sequencing data from 46,906 patients who underwent Memorial Sloan Kettering-Integrated Mutation Profiling of Actionable Cancer Targets (MSK-IMPACT) testing, the study found a notable enrichment of CH-positive patients among those with P/LP germline mutations. Specifically, a significant association was identified between P/LP germline variants in the *ATM* gene and the presence of CH. The analysis of germline and CH comutation patterns in genes such as *ATM*, *TP53*, and *CHEK2* suggested that biallelic inactivation might play a crucial role in clonal expansion. Interestingly, CH mutations in *PPM1D* were found to be depleted in patients with P/LP germline mutations in DNA damage response (DDR) genes like *ATM*, *CHEK2*, and *TP53*. Furthermore, patients with solid tumors carrying both P/LP germline mutations and CH mutations, along with mosaic chromosomal alterations, may be at heightened risk for developing secondary leukemias. Notably, germline variants in *TP53* emerged as an independent risk factor for secondary leukemias, with a hazard ratio of 36 (P < 0.001). These findings suggest a significant interplay between inherited variants and CH mutations within DDR genes, highlighting the potential for improved clinical surveillance for CH and associated comorbidities in cancer patients with these germline mutations [642].

Pourebrahim and colleagues have recently explored the intricate relationship between MDM2 haploinsufficiency and *TP53* mutations in AML. The findings reveal allele-specific loss of MDM2 in *TP53*-mutant AML, indicating that MDM2 haploinsufficiency collaborates with mutant *TP5P3* to promote myeloid-biased hematopoiesis and enhance AML predisposition, independent of p53's regulatory functions. The study demonstrates how the timing of p53 mutation significantly influences whether AML or lymphoma develops. One model induces both mutant p53 and MDM2 haploinsufficiency during early development, highlighting MDM2's critical role in hematopoiesis, while the second model mimics CH by introducing mutant p53 in adult hematopoietic stem cells (HSCs). This approach illustrates how age-related changes in HSCs interact with mutant p53, favoring myeloid transformation over lymphoma. Additionally, the research uncovers a p53-independent mechanism through which MDM2 regulates the mevalonate pathway, suggesting that targeting this pathway could enhance the efficacy of MDM2 inhibitors in AML treatment. Overall, the study provides valuable insights into the cooperative effects of HSC age, *TP53* mutations, and MDM2 haploinsufficiency, paving the way for more targeted therapeutic strategies for *TP53*-mutant AML and deepening our understanding of hematopoietic malignancies [641].

LH characterizes a rare and aggressive subtype of B-ALL with poor prognosis. In a recent study investigating the genomic landscape of LH-ALL in adults, researchers analyzed copy-number aberrations, loss of heterozygosity, mutations, and cytogenetic data in a prospective cohort of 591 Philadelphia (Ph)-negative B-ALL patients aged 18 to 84 years. This analysis identified 80 cases of LH-ALL (14%), and genomic assessment was instrumental in detecting low hypodiploidy that had been overlooked by traditional cytogenetics. The prevalence of LH-ALL increased markedly with age, ranging from 3% in younger patients (under 40) to 32% in those over 55. A key finding was the near-universal presence (98%) of somatic TP53 biallelic inactivation in adult LH-ALL cases. Additionally, TP53 mutations were detected in 34% of posttreatment remission samples, pointing to a preleukemic, multilineage TP53-mutant clone, resembling age-related clonal hematopoiesis [643]. This study underscores the link between aging and LH-ALL, highlighting TP53-mutant clonal hematopoiesis as a potential preleukemic reservoir driving aneuploidy and the development of B-ALL in older adults.

A recent study investigated the impact of cancer therapy on the development and progression of CH focusing on the evolution of TP53 mutations following chemoradiation in patients with esophageal or lung cancer. Using error-corrected duplex DNA sequencing on blood samples collected before and after treatment, the researchers tracked changes in mutation counts and clone sizes over time. Among 29 patients (median age 67) with a median follow-up of 3.9 years, the most frequently mutated genes were *DNMT3A, TET2, TP53, and ASXL1*. A significant two-fold increase in the number of *TP53* mutations was observed post-treatment, which was unique compared to other genes. Additionally, 38% of *TP53* clones increased in size, while only 5% decreased. Importantly, patients with increased *TP53* mutations following chemoradiation experienced significantly shorter overall survival. These findings reveal that exposure to cancer therapies not only contributes to the expansion of *TP53* CH mutations but also associates this expansion with poorer clinical outcomes, highlighting the potential for TP53 mutation monitoring as a prognostic marker in cancer patients undergoing chemoradiation [644].

Accordingly, Eder et al. presented a case of a 64-year-old man diagnosed with large B-cell lymphoma who experienced two relapses after standard-of-care therapy. Due to ongoing cytopenia, next-generation sequencing (NGS) revealed the presence of a small TP53-mutated clone. As a third-line treatment, the patient received CAR-T cell therapy, which led to complete remission. However, this therapy also triggered the expansion of the TP53-mutated clone, resulting in therapy-related myelodysplasia characterized by a complex aberrant karyotype. This case serves as a paradigmatic example of clonal hematopoietic progression in a patient undergoing CAR-T cell therapy, particularly in the context of a *TP53*-mutated clone, highlighting the importance of monitoring clonal evolution post-treatment [645].

Overall, mutations in genes like TP53, DNMT3A, and ASXL1 are linked to worse outcomes and higher risks of therapy-related complications. CH can act as a preleukemic reservoir, particularly in older individuals, and its expansion is often triggered by cancer treatments like chemoradiation and CAR-T therapy. Therefore, since CH plays a critical role in the development and progression of hematologic malignancies, monitoring CH in patients with hematologic malignancies could improve prognosis and guide treatment decisions, making it an essential factor in clinical practice.

## p53-based targeted therapies

### Potential p53-based gene therapy for hematologic malignancies

Gene therapy targeting the p53 tumor suppressor gene has emerged as a promising approach for cancer treatment, including hematologic malignancies. This therapy can be delivered via viral and non-viral systems, and either of them has some pros and cons [646, 647].

In recent years, gene therapy has regained popularity, and the first gene therapy approved for clinical use is based on p53. This therapy, called Gendicine, is a recombinant human p53 adenovirus created by Shenzhen SiBiono GeneTech, and it was authorized in 2003 by the China Food and Drug Administration (CFDA) for the treatment of head and neck squamous cell carcinoma (HNSCC). Thousands of patients in China have received Gendicine, and when combined with chemotherapy or radiotherapy, it has been found to produce significantly better response rates than standard treatments [29, 648, 649]. Although Gendicine is primarily used for solid tumors, its success in restoring p53 function opens the door for exploring similar therapeutic strategies in hematologic malignancies, where p53 mutations play a pivotal role in disease progression and chemoresistance.

**Fig. 4:**
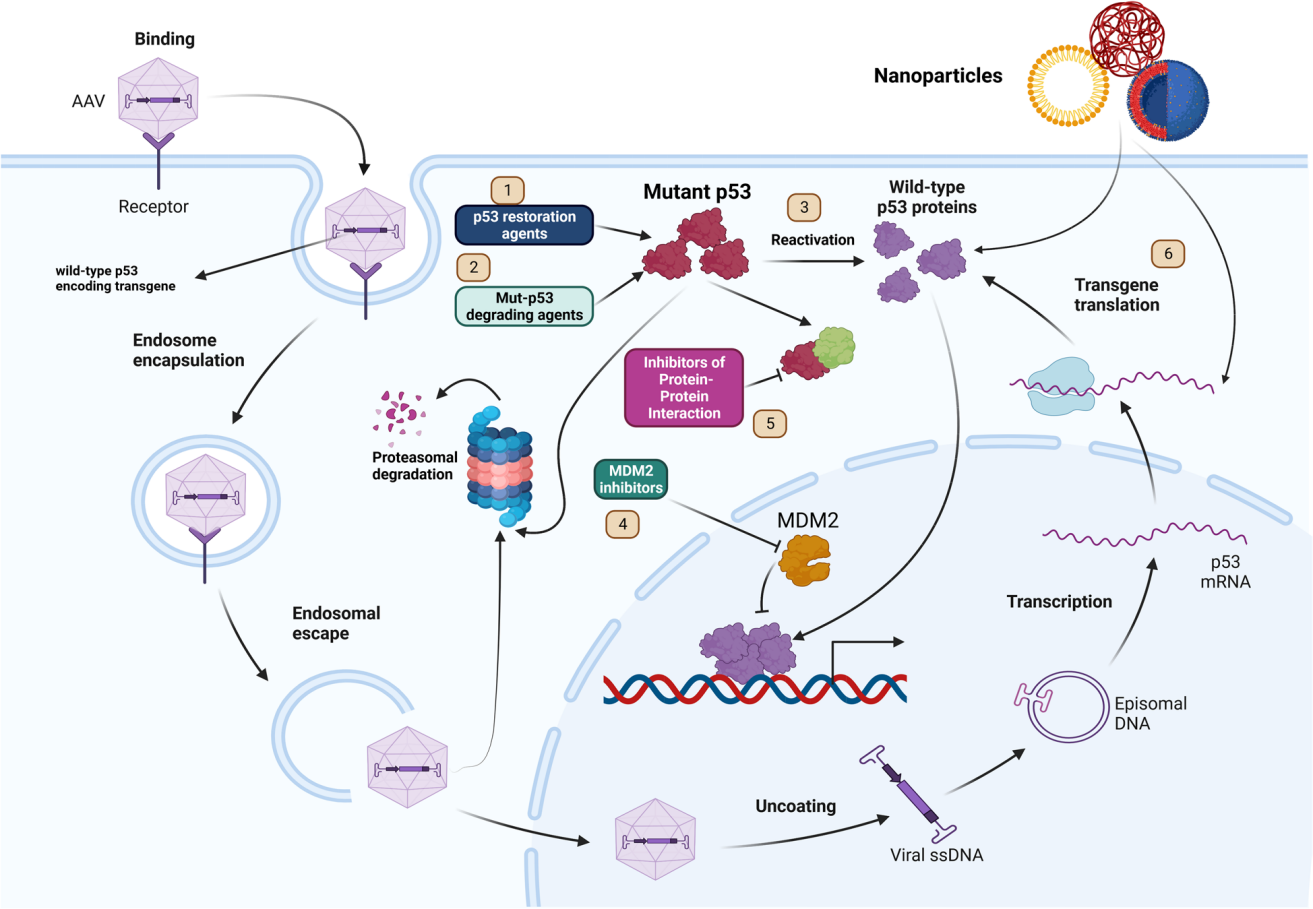
In cancer cells lacking functional wild-type p53, gene therapy approach using viral vectors and nanoparticles can introduce healthy *p53* gene to cells to induce DDR. In cases with mutant p53, resorting and reactivating agents can turn mutant p53 into wild-type form. Some agents are capable of impeding the interaction of mutant p53 with other proteins and some can propel it toward degradation, which all reduce its oncogenic activity. Moreover, MDM2 inhibitors are used to enhance the activity of wild-type p53

In recent years, nanoparticles have been studied as a means of delivering p53 gene therapy (as shown in (Fig. 4). Unlike viruses, nanoparticles are less prone to inhibitory antibodies due to their low immunogenicity, which helps prolong their circulation time and minimize immune-related side effects. Additionally, intravenous administration of nanoparticles is a more suitable approach for treating distant metastases than intratumoral injection, which is the primary method used for administering Gendicine [650, 651]. For instance, SGT-53, a cationic liposome developed by SynerGene Therapeutics, was able to sensitize glioblastoma cells to temozolomide both in vitro and in vivo by carrying w*TP53*-encoding DNA that specifically targets tumor cells via an anti-transferrin single-chain antibody fragment [652]. While SGT-53 and similar therapies have primarily been tested in solid tumors, the underlying mechanisms of p53-driven apoptosis and cell cycle regulation are highly relevant to hematologic cancers such as leukemia and lymphoma [653, 654]. Studies showing that nanoparticle-based p53 gene delivery can restore tumor suppressor functions provide a strong rationale for adapting these approaches to blood cancers.

Different types of nanoparticles, such as liposomes, polymers, and metallic nanoparticles, have been explored for p53 gene delivery [654]. Liposomal vectors like DOTAP/DOPE and cholesterol formulations, used in ovarian cancer models, effectively transported the p53 gene into cells, inducing tumor growth suppression and restoring apoptosis. Similarly, DP3-p53 liposomal formulations have been shown to induce cell cycle arrest and increase survival in animal models by delivering the p53 gene into cancerous cells [654].

Polymer nanoparticles (e.g., PLGA) are also used in p53 gene delivery, providing sustained gene expression in cancer cells. These nanoparticles have shown greater antiproliferative effects in breast cancer cells, especially when compared to other transfection agents, and are known for their biocompatibility and potential for sustained therapeutic impact [654, 655]. Moreover, metallic nanoparticles, such as gold nanoparticles conjugated with p53, are being tested to target specific cancer cells, like ovarian cancer cells, by exploiting overexpressed surface receptors such as EGFR. These nanoparticle systems offer precise targeting and have shown promising results in preclinical models. Nanoparticles also show potential for combination therapies, such as delivering both chemotherapy and gene therapy simultaneously. For instance, PLGA-based systems can encapsulate doxorubicin along with p53 gene constructs, providing a dual attack on cancer cells [654].

Recent studies have explored nanoparticle-based therapies that target p53 mutations, addressing both chemoresistance and immunosuppression. In non-small cell lung cancer (NSCLC), p53 mutations contribute to cisplatin resistance, a key challenge in treatment. Researchers developed Fluplatin, a prodrug combining cisplatin and fluvastatin, encapsulated in PEG–PE nanoparticles. Fluplatin degrades mutant p53 (mu*TP53*) and induces endoplasmic reticulum stress, significantly enhancing tumor suppression and reversing cisplatin resistance [656].

In another study administration of p53 mRNA using redox-responsive nanoparticles led to a substantial reduction in the viability of p53-null lung cancer cells and a marked decrease in tumor size in mouse models of hepatocellular carcinoma (HCC) and NSCLC [657]. Additionally, it has been shown that in HCC, a CXCR4-targeted p53 mRNA nanoparticle platform restores p53 function. When combined with immune checkpoint inhibitors (anti-PD-1), these nanoparticles reverse the immunosuppressive tumor microenvironment, enhancing anti-tumor efficacy. Together, these studies highlight the potential of p53-targeted nanotherapies to overcome both chemoresistance and immune evasion in cancer [658].

Regarding p53-based genetic therapy, an option is to use synthetic small interfering RNA (siRNA) oligonucleotides to target specific mutations within p53 mRNA [230]. Researchers have demonstrated that a single base difference in the siRNA can differentiate between wild-type and mutant p53 [230]. Delivery of an mRNA targeting a specific mutant, such as p53(R248W), resulted in decreased cancer cell survival and increased apoptosis [659]. Using highly efficient delivery systems, siRNA-based therapeutic approaches seem to be a reliable resource in gene therapy [660].

A recent study has developed a promising new theranostic platform for breast cancer treatment that utilizes extracellular vesicles (EVs) derived from breast cancer cells. These EVs have been modified to carry a lipophilic triphenylphosphonium (TPP)-modified therapeutic recombinant P53 protein (TPP/P53) to specifically target breast cancer cells [661].

While nanoparticle-based therapies have demonstrated potential in targeting solid tumors by delivering the p53 gene or restoring its function, there is a significant opportunity to explore their application in hematologic malignancies. Given the crucial role of p53 in regulating cell death and proliferation, nanoparticles could offer a promising strategy for targeting mutated p53 in blood cancers such as leukemia and lymphoma, where therapeutic options are limited. Meanwhile, some studies have demonstrated the potential of p53-based gene therapy. One study demonstrated the efficacy of a lentiviral vector-based delivery system for p53 gene therapy in CLL cells. The researchers found that the treatment induced significant apoptosis in the cells [662]. Another study investigated the use of a dual-targeting lentiviral vector for p53 gene therapy in DLBCL cells. The researchers found that the treatment induced cell cycle arrest and apoptosis in the DLBCL cells, suggesting that p53 gene therapy may be a promising approach for the treatment of DLBCL [663]. A study investigated the use of a recombinant adenovirus carrying the p53 gene for the treatment of AML. The researchers found that the treatment induced cell cycle arrest and apoptosis in AML cells both in vitro and in vivo, indicating that p53 gene therapy may be a potential treatment option for AML [664].

While the majority of p53-based gene therapies have focused on solid tumors, recent studies in hematologic cancers suggest a growing potential for adaptation. A lentiviral vector-based delivery system successfully induced apoptosis in CLL cells by restoring p53 function, while another study showed that dual-targeting lentiviral vectors caused cell cycle arrest and apoptosis in DLBCL cells. These studies underscore the adaptability of p53-based gene therapies, initially developed for solid tumors, to hematologic contexts.

Furthermore, researchers showcased the use of a CRISPR/Cas9-based gene therapy approach for the treatment of B-cell lymphoma. They found that the treatment induced cell death in the cancer cells and resulted in prolonged survival in a mouse model, indicating that p53 gene therapy using CRISPR/Cas9 may be an effective treatment for B-cell lymphoma [665]. Compared to older methods, these recent studies have demonstrated enhanced therapeutic effects and reduced toxicity in normal cells. While further research is needed to fully understand the potential of p53 gene therapy, these findings are encouraging and offer hope for patients with leukemia and lymphoma [666].

Although p53-targeted therapies are more developed for solid tumors, their underlying mechanisms of restoring tumor suppressor functions are equally relevant to hematologic malignancies. The success of Gendicine and nanoparticle-based therapies in treating cancers like HNSCC and NSCLC provides a strong foundation for exploring similar strategies in blood cancers. Given the critical role of p53 in hematologic malignancies, particularly in disease progression and resistance to therapy, there is a compelling rationale for further investigation into p53-based gene therapy and nanoparticle delivery systems in these contexts. Expanding the application of these therapies to hematologic malignancies could address significant therapeutic gaps and improve outcomes for patients with leukemia and lymphoma.

### p53-based immunotherapy

Adoptive cell transfer (ACT) is a form of cancer immunotherapy that has displayed remarkable clinical benefits for advanced cancers [667]. There are various types of ACTs, including tumor-infiltrating lymphocytes (TILs), T cell receptor (TCR) T cell transfer, and chimeric antigen receptor T (CAR-T) cells. TILs entail the process of extracting T cells from cancer patients, cultivating them in significant quantities outside the body, and subsequently reintroducing them back into the same patient to combat cancer. In the initial clinical trials for metastatic melanoma patients, adoptive transfer of ex vivo-produced autologous TILs yielded an objective response rate of 34%. However, the median duration of response was merely 4 months, despite some patients demonstrating complete responses [667]. In other research, the utilization of ACT alongside TILs as a component of a lymphodepleting treatment plan has demonstrated significant positive outcomes in clinical trials. These trials revealed that approximately 40% to 72% of patients diagnosed with metastatic melanoma achieved objective clinical responses. Among those patients, up to 40% experienced complete responses that persisted for as long as 7 years[668]. TCR-ACT, on the other hand, clones TCRs from T cells that recognize tumor antigens and then uses viruses to modify T cells, which now can specifically target tumor cells and mediate tumor regression when reintroduced to the patient [668]. CAR-T cells, unlike TCR-T cells, have both antigen binding and T cell stimulation modules engineered into their TCR. The Food and Drug Administration (FDA) has approved six CAR T-cell therapies to treat various types of cancer. Kymriah is approved for pediatric and young adult patients with relapsed or treatment-resistant ALL. Yescarta, Kymriah, and Breyanzi are approved for adults with large B-cell lymphoma that resisted two or more therapies or relapsed. Yescarta is also approved for adults with follicular lymphoma that did not respond to previous treatments. Tecartus is indicated for adults with treatment-resistant or relapsed mantle cell lymphoma. Abecma and Carvykti are approved for adults with multiple myeloma that did not respond to at least four prior therapies. These innovative CAR T-cell therapies offer promising treatment options for patients with challenging and aggressive blood cancers [669].

One critical aspect of generating effective TCR-Ts or CAR-Ts involves cloning or engineering a TCR that specifically targets neoantigens from tumors. Neoantigens are antigens that originate from mutated proteins within tumor cells. Mutated p53 proteins are promising candidates for generating neoantigens due to their high expression levels in tumors. In 1979, Levine et al. discovered that sera from mice with transformed tumors contained antibodies against endogenous p53, making it the first study to demonstrate the immunogenicity of the p53 protein and its ability to activate T helper cell response [670]. Subsequent research revealed that both human and murine WT p53 and certain p53 mutants can activate cytotoxic T cells, although these studies were conducted under non-endogenous conditions [671]. Thus, the activation of T-cell responses by endogenous mutant p53 in human tumors and the immunogenicity of peptides containing p53 mutations have yet to be fully understood.

A recent scientific inquiry delved into how the immune system responds to mutant p53 in 140 patients with different types of tumors. The study revealed that endogenous p53 mutants can instigate the activation of CD4 + and CD8 + T cells. What's interesting is that 39% of the patients who underwent testing had Tumor-Infiltrating Lymphocytes (TILs) that could identify self-produced mutant p53 neoantigens. Furthermore, isolated TILs or TCR-engineered T cells from patients could recognize cancer cells that expressed endogenous p53 mutants [672]. While the research did not demonstrate the effectiveness of TILs or TCR-engineered T cells in reducing tumor size, it did provide strong proof that endogenous p53 mutants in human tumor cells are immunogenic, which opens doors for using mutant p53-based ACT therapies. However, it's worth noting that not all peptides that have *TP53* mutations are immunogenic, likely due to sequence requirements for neoantigens. Peptides that have hotspot mutations such as R175H, Y220C, G245S, R248Q, R248W, and R282 can activate T cell responses, but with different frequencies [672].

The immune checkpoint blockade is a type of cancer immunotherapy that aims to prevent tumors from evading the body's immune system. Tumors have various ways of avoiding detection by immune cells like T cells or natural killer cells. One such mechanism involves increasing the presence of molecules that are part of the immune checkpoint, like PD-L1 on tumor cells. PD-L1 inhibits the activation of T cells and triggers their death. However, drugs that block the interaction between PD-L1 and its receptor, PD-1, have been shown to be highly effective in treating cancer [673]. In fact, the FDA has approved several PD-L1 or PD-1 blocking antibodies for cancer treatment.

A recent study found that when p53 is activated, either through genotoxic or non-genotoxic stress, it triggers the expression of PD-L1 and PD-1 in multiple cancer cell lines in vitro. However, the actual impact of this discovery on cancer treatment remains uncertain [674]. On the other hand, an independent study revealed that p53 decreases the expression of PD-L1 by inducing microRNA 34a (miR34a). miR34a binds to the 3' untranslated region (UTR) of PD-L1, which reduces its expression. This implies that p53 plays a role in regulating immune checkpoints [675]. A phase I clinical trial involving the use of liposomes containing miR34a showed that the agent enhanced cytotoxic CD8 + T cells while reducing the CD8 + ; PD-L1 + subpopulation. This suggests that the p53-miR34a-PD-L1 axis could be used to inhibit PD-L1. Additionally, it's worth noting that the miR34 family of molecules has numerous targets, including CDK4, RUNX2, and other pro-oncogenic or survival factors. Therefore, activating the p53-miR34 axis could simultaneously inhibit PD-L1 and several oncogenic pathways [675].

Recent research aimed to investigate whether *TP53* and KRAS mutations could serve as predictors for the response of lung adenocarcinoma to PD-L1 blockade. The findings of the study indicate that the presence of *TP53* or KRAS mutations is associated with higher PD-L1 expression and a better response to PD-L1 blockade [676]. Moreover, a thorough analysis of publicly available clinical trial data revealed that the co-occurrence of *TP53* and KRAS mutations is a dependable predictor of the response of lung cancer to PD-L1 blockade. Despite the unclear mechanism underlying this correlation, these studies suggest that *TP53* mutation status has the potential to be a clinical biomarker for guiding immune checkpoint blockade therapy. However, further research is necessary to elucidate the precise connection between *TP53* mutation status and immune checkpoint blockade therapy response [676].

All in all, the p53-based immunotherapy approach has shown promise in inhibiting the PD-L1 immune checkpoint in various solid tumors. However, it has not been tested on hematologic malignancies. Nevertheless, given the observed effects on immune checkpoint regulation and the potential of this approach to inhibit several oncogenic pathways simultaneously, p53-based immunotherapy may hold significant therapeutic potential for the treatment of hematologic malignancies. Further research is necessary to determine the safety and efficacy of this approach in the treatment of these types of cancers.

### Reactivating the wild-type function of mutant p53

Following the identification of p53 as a quintessential tumor suppressor and p53-related mutations, meticulous studies have helped obtain a more thorough understanding of reviving both wild-type p53 and mutant forms of it. Hence, small molecules-mediated activation of wild-type p53 or restoring mutant p53 function by them has provided a plethora of therapeutic options for anti-cancer therapy [677]. Since there are various types of p53-restoring small molecules with different mechanisms of action, Liu et al. have proposed criteria, including the binding capability to mutant p53, thermostabilizing, antibody (e.g., PAb1620)-mediated visualization of the conformational change of mutant p53 to wild-type form, and up-regulation of genes targeted by p53, which fulfilling them signifies the compound to be a p53 reactivating agent [678]. The main plot of these compounds is to stabilize the natural structure of the p53 DBD to resurrect its DNA binding ability and trans-activation of p53 target genes such as CDKN1A in order to induce cell death followed by obliterating the tumor [679]. Despite all the progress in this field, the exact mechanism of mutant p53 refolding by small molecules is yet to be discovered.

CP‑31,398 is the first identified small molecule capable of reactivating mutant p53 [680], which binds precariously to a thiol group of p53 DBD [681]. Refolding the mutant p53, and barricading p53 ubiquitination as well as protecting wild-type p53 from thermal denaturation appear to be CP-31398 primary mechanism of action, although it can induce DNA damage as well [680, 682]. The anti-tumor activity of CP-31398 has been reported to be effective in cancerous cell lines and mice with colon carcinoma, melanoma, hepatocellular carcinoma, esophageal carcinoma, endometrial cancer, and urothelial cancer of the bladder [680, 683–685]. A most recent study also proclaimed that CP-31398 is able to augment the cytotoxic lymphocyte-mediated elimination of breast cancer cells via p53-dependent autophagy [686]. Furthermore, results of combining CP-31398 with an MDM2 inhibitor have delineated a considerable debilitation in endometrial cancer cell invasion, metastasis, and resistance to apoptosis in a p53-dependent mode [687]. Not only could this combination work on cervical cancer, but it also appears to be a robust approach to mesothelioma, irrespective of the p53 genotype [688]. Moreover, the overproduction of reactive oxygen species (ROS) in multiple myeloma has been shown to provoke an apoptotic response in MM cells [689]. Accordingly, a study reported that CP-31398 could also exert apoptosis in a p53 -p53-independent manner in which CP-31398 excessively elevates ROS in both MM cell lines and MM xenografts in mice [690].

In 2002, Bykov and colleagues identified PRIMA‑1, a low molecular weight compound capable of restoring p53 mutant (R273H) to its native functional mode [691, 692]. PRIMA1 and its more potent and methylated form (APR-246) act through targeting cysteine residues of p53 (Cys-124 and -277), leading to its refolding [693, 694]. As a result, p53 would express its target genes, including *CDKN1A, PUMA*, and *BAX,* in tumor cells [691, 695, 696]. The enhanced APR-246’s lipophilicity and cell permeability give it more potential than PRIMA1 [695]. Although these agents are pro-drugs, a hydrolysis-mediated process turns them into their active form, termed methylene quinuclidinone (MQ) [696]. PRIMA‑1 and APR‑246 have been shown to possess tumor-suppressive features on cells from diverse origins that express mutant p53, for example, sarcomas, mammary carcinomas, fibrosarcomas, small cell lung cancer, oesophageal adenocarcinoma and breast cancer [691, 697–699]. The APR-246 low toxicity has been reported through conducting phase I/II clinical trials on various hematological malignancies, lymphomas, and prostate cancer [700]. In the study, p53 upregulation betokened anti-tumor activity of APR-246, and amongst all, blast reduction in the bone marrow of AML patients was more notable [701]. According to the evidence, cases with wild-type p53 manifested no clinical response consistent with the predefined response criteria [701, 702].

The *TP53* mutation indicates the most unfavorable prognostic criteria in CLL patients, and recent Bruton´s tyrosine kinase inhibitors such as Ibrutinib have shown earlier disease progression [703]. In this regard, Jaskova et al. reported the effectiveness of APR-246 in evoking an apoptotic response in CLL cells with mutated *TP53*, irrespective of baseline p53 level [704]. However, further clinical trials Using APR-246 in combination with other drugs, including Ibrutinib, Venetoclax, and Azacitidine on hematological malignancies and lymphomas, have been initiated [NCT04214860], [NCT04419389] [700]. Not only is APR-246 a potential p53 -restoration agent, but also treating acute promyelocytic leukemia-derived cells (NB4 Cell line) with PRIMA-1 has demonstrated to effectively induce caspase-mediated apoptosis through sabotaging nuclear factor-κB and downregulating c-Myc, XIAP, and Bcl-2. In which case, PRIMA-1 preferentially affected cancer cells, and healthy cells seemed intact [705].

MIRA‑1 and STIMA‑1 are small molecules that selectively affect tumor cells with mutated *TP53* and induce p53 target genes [681, 706]. Like CP-31398, both MIRA-1, and STIMA-1 react with cysteine residues of mutant p53 leading to the conformational transition into p53 native-like form [707]. Thus, a study demonstrated MIRA-1’s capability of inducing p53-mediated apoptosis in different tumor cells in which each had borne a discrete mutation (R175H, R248Q, and R273H) in their p53 gene [706]. The study also showcased in vivo activity of MIRA-3 upon SCID mice bearing mutant-p53 tumor xenografts. Albeit it was potential as well as MIRA-1, but a higher rate of toxicity had narrowed its therapeutic application [706]. The utilization of MIRA-1 on MM cell lines, primary samples, and mouse xenograft model initially showed elevated apoptotic response irrespective of p53 status following down-regulation of Mcl-1 and c-Myc and up-regulation of Puma and Bax. Furthermore, even removing p53 from the equation by knocking it down had not revoked the MIRA-1-induced apoptosis, which indicated an additional p53 -independent apoptotic pathway mediated by MIRA-1 [708]. Not only MIRA-1 alone had not showcased any particular toxicity on MM cells but also combining it with dexamethasone, doxorubicin, or Bortezomib has presented a synergetic outcome [708]. STIMA-1, a derivate of CP-31398, could as well induce p53 -mediated apoptosis in different tumors with mutated *TP53*, including p53 -R175H and p53 -R273H in lung carcinoma and osteogenic sarcoma cell lines, respectively [681]. Moreover, MIRA-1 [706] and STIMA-1 [681], as well as APR-246 [695] and PK11007 [709], are able to exert their anti-tumor potential not only through restoring mutant p53 but also via targets, such as cellular redox regulators. More recently, a hybrid compound called KSS-9 represented a similar mechanism of action in a reciprocal reduction of mutant p53 and generation of native-like p53 [710].

Nearly 30% of p53-related mutations volatile the DBD, resulting in temperature sensitivity, in which the T_m_ might become lower than the body temperature and could cause p53 denaturation [10, 711]. Drug-mediated thermostabilization of mutated forms of p53 (R175H, Y220C, G245S, R249S, and R282) by two thiol alkylating agents, namely 3-benzoyl-acrylic and (E)-4-(4-fluorophenyl)-4-oxobut-2-enoic acid, have been described to rise T_m_ of the mutant p53 up to 3°C, which empowers the p53 to bind DNA [712]. Furthermore, it was explicated that PK11007 (2-sulfonyl pyrimidine) is also capable of thiol alkylation and thermostabilizing mutant p53 without interfering with its DNA binding [709]. In addition to aforementioned small molecules, there is a vast variety of agents such as Zn^2+^ chelators (e.g., ZMC1 [709] and COTI‑2 [713]), peptides (e.g., pCAPs [714] and Reacp53 [714]), RETRA [715], Stictic acid [693] and Chetomin [716] that could be used for p53 restoration, as well. Furthermore, all p53 restoring agents have been summarized in Table 5.

### Stabilizing p53 in wild-type p53-expressing cancer cells through inhibition of the Mdm2–p53 interaction

Overexpression of MDM2, discovered in various human tumors, dramatically diminishes p53 function [717]. Another p53 restoration approach is to restrict p53-MDM2 interaction, in addition to direct inhibition of MDM2, blocking MDM2-related sites on p53 by particular compounds can also hinder the MDM2-p53 interplay. One such agent is RITA that has been shown to be competent in preventing MDM2-mediated degradation of p53 in multiple tumor cell lines both in vivo and in vitro [718]. Additionally, p53 interaction with other negative regulators can also be abolished by RITA, which all in all results in a significant apoptotic response [718]. The anti-tumor and pro-apoptotic effects of RITA prerequisites eIF2α phosphorylation in a way that inhibition of eIF2α hampers its effects [719]. The effects of RITA on leukemia and multiple myeloma cell lines bestows enhanced p53-dependent apoptosis [720, 721]. Importantly, it has been determined that applying RITA in combination with doxorubicin upon the pre-B ALL cells could promote chemosensitivity accompanied by p53-dependent and p53-independent apoptosis [721]. Similar outcomes have also been achieved on colorectal cancer cells in which RITA was used in combination with chemotherapeutic agents such as 5-Fluorouracil and Oxaliplatin [722]. RITA also applies its effects through inducing p53-independent apoptosis; for instance, it can provoke an apoptotic response in the p53-null CML cell line by inhibiting STAT5, Akt, and NF-κB signaling pathways along with downregulating proliferative genes such as c-Myc [723]. Additionally, a thorough study upon 16 cell lines harboring different p53 status elucidated that RITA can exert its apoptotic effects not only on wild-type 53 or mutant p53 but also in p53-null cells in a caspase- and BAX/BAK-dependent manner via activation of JNK/SAPK and p38 [724].

Nutlin, first identified by Vassilev et al., are potential MDM2 inhibitors that can interact with the p53-binding pocket of MDM2, which as a consequence, results in p53 accumulation and p53 -mediated cycle arrest and apoptosis in cancer cells [725]. Nutlin-3 has drawn more attention compared to other nutlins due to its higher specificity in activating and stabilizing the p53 pathway [725]. Nutlins such as RG7112, MI 773, DS 3032b, NVP-CGM097, SAR405838, MK-8242, AMG 232, and RG7388 are under phase I/II clinical trials to be tested on a broad spectrum of diseases, including hematologic neoplasms and solid tumors [27, 726, 727]. Several studies on different hematological malignancy cells, including AML, CLL, ALL, MM have designated that nutlins affect wild-type p53 in transcription-dependent [728–730] or transcription-independent manners [731–734]. Moreover, using nutlin and Bcl-2 (anti-apoptotic protein) inhibitors concomitantly in AML cells have shown induced synergistic apoptosis. It is worth mentioning that the cells in the G1 phase are mostly shielded from nutlin-induced apoptosis, whereas Bcl-2 inhibitors can override this obstacle and induce apoptosis in G1 [735]. Likewise, compounds such as PRIMA-1, PK7088, RITA, and MI‑773 have been shown to promote nutlin effects synergistically [678, 726, 736, 737].

Recently, it has been found that the p53-induced senescence mediated by nutlin may help with skewing macrophage polarization to its antitumor M1 state. It has been elucidated that tumor cells expressing wild-type p53 can secrete mediators that aid this process and mark themselves with opsonization signals. In which case, macrophages might be able to invade cells influenced by nutlin-mediated p53-induced senescence [726, 738]. Intriguingly, p53-defected cells secrete factors that promote macrophages (M2) in favor of tumors [738]. Since MDM2 inhibitors have made it through to clinical trials, and the results are promising so far, it could be elicited that the future for these inhibitors to be used in the clinic is bright.

The development of MDM4 inhibitors has been more challenging due to the structural similarity between MDM2 and MDM4, as well as the fact that MDM4 is less abundant than MDM2. Nonetheless, several MDM4 inhibitors have been developed, including idasanutlin (RG7388), ALRN-6924, and AMG-232 [739]. Idasanutlin has been evaluated in preclinical studies for the treatment of AML and has shown promising results, particularly in combination with cytarabine. ALRN-6924 is a dual inhibitor of MDM2 and MDM4 that has shown activity in preclinical studies for the treatment of AML and lymphoma [739]. AMG-232 has also shown activity against both MDM2 and MDM4 in preclinical studies and is currently being evaluated in clinical trials for hematologic malignancies [739]. MDM2 and MDM4 inhibitors are promising therapeutic strategies for the treatment of hematologic malignancies, particularly AML. More research is needed to optimize dosing and identify patient populations that are most likely to benefit from these treatments. Additionally, combination therapies with other agents may enhance the efficacy of MDM2 and MDM4 inhibitors and improve outcomes for patients with hematologic malignancies.

Currently, several structurally distinct small-molecule MDM2 inhibitors have progressed to clinical trials, yet they face substantial setbacks [740]. Notable candidates such as RG7112, RG7388, and AMG232, which showed strong anti-tumor activity in preclinical studies, have demonstrated limited efficacy in clinical settings. RG7112, despite its promising preclinical results, yielded no significant tumor response in clinical trials and caused serious adverse reactions like neutropenia and thrombocytopenia in nearly half of the patients. Similarly, RG7388 failed to induce significant p53 activation and resulted in dose-dependent toxicity. AMG232 encountered drug resistance due to increased mutation frequency in circulating cell-free DNA (ccfDNA), including the emergence of new p53 mutations. The key issues identified include: a mismatch between preclinical and clinical efficacy, hematological toxicity caused by p53 activation in bone marrow, and the limitation of MDM2 inhibitors to tumors with wild-type p53, with resistance arising from induced p53 mutations. These challenges highlight the need for novel strategies and optimized dosing regimens in ongoing clinical research [741].

### Targeting truncated p53

Most of the *TP53* mutations associated with cancer are missense mutations, but about 10% of *TP53*-mutated tumors have nonsense mutations [134]. These mutations result in the production of shortened p53 proteins that are usually broken down quickly by the nonsense-mediated mRNA decay mechanism. Since these truncated proteins have a short lifespan and lack most of the p53 protein sequence, efforts to restore their function using the aforementioned methods are likely to be ineffective [134].

There are two proposed methods for activating the p53 signaling pathway in cancer cells with p53-truncating mutations. The first approach involves using molecules that promote translational readthrough, allowing the translation machinery to produce full-length p53 protein by bypassing RNA stop codons. Examples of such compounds include gentamicin, G418, and NB124, which can rescue the production of functional p53 and promote cancer cell death [134, 742]. The second approach involves inhibiting the nonsense-mediated mRNA decay (NMD) process, which can be achieved by using drugs like NMD14 that target SMG7, a key component of the NMD machinery [743]. Although drugs like ataluren have already entered phase III clinical trials for cystic fibrosis, their effectiveness as anticancer agents for tumors with *TP53* nonsense mutations is still uncertain. Additionally, these compounds are highly toxic, which raises questions about their suitability as selective p53-targeted drugs. There is currently limited information available regarding the use of NMD14 specifically for hematologic malignancies. As NMD14 targets SMG7 it has the potential to be effective in cancers that harbor *TP53* nonsense mutations, which are commonly found in hematologic malignancies [744]. However, more research is needed to determine the efficacy and safety of NMD14 for the treatment of hematologic malignancies.

Overall, while the majority of p53-targeting therapeutic approaches have been predominantly studied and utilized in the context of solid tumors, several studies have demonstrated their potential application in hematologic malignancies. The success of compounds such as APR-246, CP-31398, and MIRA-1 as well as gene therapies and immunotherapies in preclinical and early clinical settings for blood cancers, along with ongoing clinical trials (Table 6), is a testament to their promise in treating hematologic malignancies. These developments underscore the potential of these therapies to be incorporated into the treatment landscape for blood cancers, warranting further investigation and exploration to fully harness their therapeutic capabilities in this domain.

**Table 6:** Overview on p53 targeted therapies

Therapeutic Approaches	Targeting Compounds	Targeted mutant types	Clinical Trials		Ref
**Reactivation and Restoration**	PRIMA-1/PRIMA-1MET (APR-246)	R175H/ R273H	NCT03588078	MDS/ MPN/ AML/ CML	[745]
			NCT03072043	MDS/ MPN/ AML/ CML	
			NCT04990778	Recurrent Mantle Cell Lymphoma/Refractory Mantle Cell Lymphoma	
			NCT02999893	Oesophageal Carcinoma	
	HO-3867	R175H/ R273H/ G245S/ R248W/ R249S	-	-	[746, 747]
	ZMC1/NSC319726	R175H/ G245S	-	-	[748, 749]
	MIRA-1	R175H/ R273H/ R248Q/ R248W/ R282W	-	-	[750]
	STIMA-1	R175H/ R273H	-	-	[681]
	KSS-9	R175H	-	-	[710]
	MIRA-1	R175H/ R273H/ R248Q/ R248W/ R282W	-	-	[750]
	Flavokawain B	R273H			[751]
	COTI-2	R175H	NCT02433626	Cancers	[752]
	C85	R175H	-	-	[753]
	SLMp53 -1	R175H/ R273H/ Y220C/ R248Q/ R248W/ R282W	-	-	[754, 755]
	PEITC*	R175H	NCT01790204	Oral Cancer	[756]
	SCH529074	R175H/ R273H/ R248W/ R249S	-	-	[757]
	Stictic acid	R175H	-	-	[758]
	pCAPs*	R175H/ R249S	-	-	[714]
	CDB3	R175H/ R273H/ G245S/ R249S	-	-	[759–762]
	Chetomin	R175H	-	-	[716]
	Ellipticine	R175H/ R273H/ R249S	-	-	[763]
	p53 R3	R175H/ R273H/ R248W	-	-	[764]
	PK11007/ PK083/ PK7088/ PK5196	Y220C	-	-	[678, 709, 765, 766]
	CP-31398	R273H/ R249S	-	-	[767–769]
	HBAP	R273H	-	-	[770]
	Curcumin	R273H	NCT01948661	Colorectal Adenoma	[751, 771, 772]
			NCT02944578	Neoplasms	
	Alpinetin	R273H	-	-	[751]
	MANIO	R175H/ R273H /G245S/ R248Q/ R248W/ R282W	-	-	[773]
**Impeding of Protein–Protein Interaction**	Statins	R175H/ Y220C/ R248W	NCT03560882	Hematologic and solid tumors	[774]
			NCT04767984	Colorectal Carcinoma/ Ulcerative Colitis	
			NCT03358017	Triple Negative Breast Cancer	
	ReACp53	R175H/ R248Q	-	-	[775]
	RETRA	R273H	-	-	[715]
	Prodigiosin	R273H/ R248Q	-	-	[776]
	LEM2	R273H	-	-	[777]
	ATRA	R273H	NCT00617409	Small Cell Lung Cancer	[778]
**Mutant-p53 Degradation**	Arsenic trioxide	R175H/ R273H/ R248W/ R282W	NCT04489706	Ovarian Cancer/ Endometrial Cancer	[779, 780]
			NCT04695223	Refractory Cancer/ Intractable Cancer	
			NCT03381781	AML	
			NCT03855371	AML/MDS	
			NCT04869475	Refractory Solid Tumors	
			NCT03377725	MDS	
	Gambogic acid	R175H/ R273H	-	-	[781]
	Geldanamycin	R175H/ R273H/ R248W	-	-	[782, 783]
	NSC59984	R175H/ R273H	-	-	[784]
	17-DMAG/ 17-AAG	R273H			[785, 786]
	Ganetespib	R273H/ R248Q	NCT02012192	Epithelial Ovarian Cancer/ Fallopian Tube Cancer/Primary Peritoneal Cancer	[786]
	SAHA*	R273H/ R249S	-	-	[787, 788]
	YK-3–237	R273H/ R249S	-	-	[789]
	Disulfiram	R273H	-	-	[790]
	Sodium butyrate	R249S	-	-	[791]
	Spautin-1	R282W	-	-	[792]
**Thermal Stabilizer**	3-Benzoylacrylic acid	R175H/ Y220C/ R249S/ R282W	-	-	[712, 793]
	PRIMA-1/PRIMA-1MET (APR-246)	R175H/ R273H	-	-	[745]
	PK11007/ PK083/ PK7088/ PK5196	Y220C	-	-	[678, 709, 765, 766]
	SLMp53 -1	R175H/ R273H/ Y220C/ R248Q/ R248W/ R282W	-	-	[754, 755]
	MANIO	R175H/ R273H /G245S/ R248Q/ R248W/ R282W	-	-	[773]
	LEM2	R273H			[777]
	CDB3	R175H/ R273H/ G245S/ R249S	-	-	[759–762]
**Gene Therapy**	Gendicine	Regardless of p53 mutation status	CFDA approved	Head and neck cancer	[29]
	Adenovirus SG600-p53	Regardless of p53 mutation status	-	Various cancers (Lung/liver/Cervical//Pancreas)	[794]
	Adenovirus SG635-p53	Regardless of p53 mutation status	-	Breast cancer	[795]
	Adenovirus AdSurp-p53	Regardless of p53 mutation status	-	Liver and Gallbladder cancer	[796]
	Adenovirus AdCB016- mp53 (268N)	Regardless of p53 mutation status	-	Cervical cancer	[797]
	Adenovirus AdΔ24-p53	Regardless of p53 mutation status	-	Various cancers	[798]
	VSV-p53 wt	Regardless of p53 mutation status	-	Pancreatic cancer	[799]
	VSV-p53 CC	Regardless of p53 mutation status	-	Pancreatic cancer	[799]
	Newcastle disease virus rNDV-p53	Regardless of p53 mutation status	-	Hepatoma	[800]
	Adenovirus AdRGD-PGp53	Regardless of p53 mutation status	-	Prostate carcinoma	[801]
	Adenovirus rAd-p53	Regardless of p53 mutation status	NCT02561546	Diabetes Concurrent with Hepatocellular Carcinoma	[802]
			NCT01574729	Non-small Cell Lung Cancer	[803]
			NCT00902122	Advanced Malignant Thyroid Tumors	[804]
			NCT00902083	Advanced Oral and Maxillofacial Malignant Tumors	-
			NCT00894153	Head and Neck Malignant Tumors in Advanced Stage	[805]
			NCT03544723	Solid Tumor Lymphoma	[806, 807]
	Adenovirus Ad5CMV-p53	Regardless of p53 mutation status	NCT00003450	Ovarian Cancer Peritoneal Cavity Cancer	-
			NCT00004041	Brain and Central Nervous System Tumors	[808]
			NCT00004038	Breast Cancer	-
			NCT00003167	Bladder Cancer	[809]
			NCT00064103	Lip and Oral Cavity Cancer Oropharyngeal Cancer	-
			NCT00003649	Lung Cancer	[810]
	Adenovirus rAd-p53 SCH-58500	Regardless of p53 mutation status	NCT00004080	Brain and Central Nervous System Tumors	[811]

## Conclusion and future prospectives

The development of p53-based cancer therapy is making progress, but there are still many obstacles to overcome before effective and selective drugs can be used in the clinic. Although adenovirus-based gene therapy has been approved for clinical use in China, this approach has not gained widespread use due to the limitations of adenovirus-based gene delivery in tumors. Targeted therapy for the p53 pathway has not yet received FDA approval. There are several reasons for these unsuccessful attempts. Firstly, most cancer cells have loss-of-function mutations in *TP53*, making them difficult to target with drugs. For example, drugs that restore the correct shape to mutant p53 have not progressed beyond Phase III clinical trials. Secondly, the different mechanisms and context-dependence of mutant p53's GOF complicate the design of clinical trials for targeted therapies. Although this can be overcome by identifying biomarkers to select patients who are most likely to respond to each p53-based therapeutic agent, it requires a deeper understanding of the mechanisms underlying the GOF of mutant p53 in each cancer type.

It is important to stress that p53 aberrations are commonly associated with worse prognosis, particularly in advanced stages and relapses of the disease. The frequency of these mutations varies significantly across different hematological malignancies, with some cancers exhibiting a higher incidence of p53 mutations than others. This variation in mutation frequency is a key consideration for therapeutic strategies, as it influences the disease trajectory and patient outcomes. Moreover, while p53 mutations are a hallmark of later stages of disease progression, the interplay between p53 mutations and treatment resistance remains a major challenge.

One of the main concerns is the emergence of resistance to treatment, which can occur through various mechanisms such as mutation of the *TP53* gene or activation of anti-apoptotic genes. Combination therapy and immunotherapy may be promising approaches to reduce resistance. In vivo testing of p53-based drugs is primarily conducted in mice, but there are significant differences between mice and humans, which can affect the efficacy and safety of these drugs. Advanced experimental methodologies, such as organ-on-a-chip and ex vivo models, may help to address these differences and accelerate the transition of p53-targeted drugs to the clinic. However, the biggest challenge is our limited understanding of human biology and the complex processes that occur within cancer cells when drugs are administered. Our current screening methods are improving, but we still lack sufficient knowledge to predict off-target effects and other potential adverse effects. Therefore, more definitive testing models and deeper understanding are urgently needed to ensure the safety and effectiveness of p53-based cancer therapy.

Despite over 40 years of research, it seems that we have only scratched the surface of the vast knowledge surrounding the p53 pathway. Moving forward, immunotherapy approaches based on Adoptive Cell Transfer (ACT), such as TILs, TCR-T, or CAR-T cells, hold great promise in developing effective treatments for the p53 pathway. These approaches rely heavily on the expression of neoantigens from p53 mutants in tumor cells. Consequently, their effectiveness is less influenced by the varied biology behind p53 mutants compared to other context-dependent strategies. Additionally, most *TP53* mutations are concentrated in hotspot residues within the DNA binding domain, leading to a reduction in the diversity of neoantigens from these mutations. By combining the knowledge of p53 biology with immunotherapy, there is potential to revolutionize the design of p53-based cancer therapy in the future.

## Data Availability

No datasets were generated or analysed during the current study.

## Abbreviations

DDRs: DNA damage responses
DBD: DNA-binding domain
MDS: Myelodysplastic syndrome
MM: Multiple myeloma
NGS: Next-generation sequencing
WT: Wild-type
CFDA: China food and drug administration
ChIP: Chromatin immunoprecipitation
TFs: Transcription factors
Akt: Protein kinase B
YY: Yin-Yang
NF-1: Nuclear factor 1
PDAC: Pancreatic ductal adenocarcinoma
ER: Endoplasmic reticulum
ISREs: Interferon-stimulated response elements
Btf: Bcl-2-associated transcription factor
PKCδ: Protein kinase C δ
CPE: Core promoter element
RREB-1: Ras responsive-element binding protein-1
BCL6: Proto-oncogene B cell CLL/lymphoma 6
CTCF: CCCTC-binding factor
DP: Dimerization partner
eEF1Bγ: Eukaryotic elongation factor subunit 1B gamma
lncRNA: Long non-coding RNA
PKNOX2: PBX/Knotted Homeobox 2
IGFBP5: Insulin-like Growth Factor Binding Protein 5
NeuroD1: Neurogenic differentiation factor 1
FOXD3: Forkhead box D3
DNMT1: DNA-methyl transferase 1
UTRs: Untranslated regions
HuR: Human antigen R
UV: Ultraviolet
PTMs: Post-translational modifications
TADs: Tandem activation domains
PRD: Proline-rich domain
TD: Tetramerization domain
CTD: Carboxy-terminal domain
CDKs: Cyclin-dependent kinases
LOH: Loss of heterozygosity
TCGA: Cancer Genome Atlas
SNPs: Single nucleotide polymorphisms
AML: Acute myeloid leukemia
ALL: Acute lymphoblastic leukemia
CML: Chronic myeloid leukemia
CLL: Chronic lymphocytic leukemia
MDS: Myelodysplastic syndromes
NK: Natural killer
HL: Hodgkin lymphoma
NHL: Non-Hodgkin lymphoma
DLBCL: Diffuse large B‐cell lymphoma
PB/PC-Fs: Plasmablastic/plasmacytoid features
MCL: Mantle cell lymphoma
FL: Follicular Lymphoma
HCL: Hairy cell leukemia
BL: Burkitt lymphoma
MALT: Mucosa-associated lymphoid tissue
